# DNA damage contributes to age‐associated differences in SARS‐CoV‐2 infection

**DOI:** 10.1111/acel.13729

**Published:** 2022-10-18

**Authors:** Rui Jin, Chang Niu, Fengyun Wu, Sixin Zhou, Tao Han, Zhe Zhang, Entao Li, Xiaona Zhang, Shanrong Xu, Jiadong Wang, Shen Tian, Wei Chen, Qinong Ye, Cheng Cao, Long Cheng

**Affiliations:** ^1^ Beijing Institute of Biotechnology Beijing China; ^2^ College of Life Sciences Capital Normal University Beijing China; ^3^ Department of Surgery Chinese PLA General Hospital Beijing China; ^4^ BaYi Children's Hospital, the Seventh Medical Center Chinese PLA General Hospital Beijing China; ^5^ Key Laboratory of Jilin Province for Zoonosis Prevention and Control, Changchun Veterinary Research Institute Chinese Academy of Agricultural Sciences Changchun China; ^6^ School of Life Science Anqing Normal University Anqing China; ^7^ Department of Radiation Medicine, School of Basic Medical Sciences, Institute of Systems Biomedicine Peking University Health Science Center Beijing China

**Keywords:** aging, DNA damage, telomere, viral infection

## Abstract

Coronavirus disease 2019 (COVID‐19), caused by the severe acute respiratory syndrome coronavirus 2 (SARS‐CoV‐2), is known to disproportionately affect older individuals. How aging processes affect SARS‐CoV‐2 infection and disease progression remains largely unknown. Here, we found that DNA damage, one of the hallmarks of aging, promoted SARS‐CoV‐2 infection in vitro and in vivo. SARS‐CoV‐2 entry was facilitated by DNA damage caused by extrinsic genotoxic stress or telomere dysfunction and hampered by inhibition of the DNA damage response (DDR). Mechanistic analysis revealed that DDR increased expression of angiotensin‐converting enzyme 2 (ACE2), the primary receptor of SARS‐CoV‐2, by activation of transcription factor c‐Jun. Importantly, in vivo experiment using a mouse‐adapted viral strain also verified the significant roles of DNA damage in viral entry and severity of infection. Expression of ACE2 was elevated in the older human and mice tissues and positively correlated with γH2AX, a DNA damage biomarker, and phosphorylated c‐Jun (p‐c‐Jun). Finally, nicotinamide mononucleotide (NMN) and MDL‐800, which promote DNA repair, alleviated SARS‐CoV‐2 infection and disease severity in vitro and in vivo. Taken together, our data provide insights into the age‐associated differences in SARS‐CoV‐2 infection and a novel approach for antiviral intervention.

## Graphical abstract

Our data suggest DNA damage contributes to age‐associated differences in SARS‐CoV‐2 infection and provide a novel target for anti‐viral intervention.

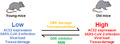

**Abbreviations**
ACE2: angiotensin‐converting enzyme 2
ATM: ataxia telangiectasia mutated
ATR: ataxia telangiectasia and Rad3‐related
BER: base excision repair
CHIP: chromosome immunoprecipitation
COVID‐19: coronavirus disease 2019
DAPI: 4′,6‐diamidino‐2‐phenylindole
DDR: DNA damage response
GCDR: gentle cell dissociation reagent
HR: homologous recombination
IR: ionizing radiation
NHEJ: nonhomologous end joining
NMN: nicotinamide mononucleotide
ODM: organoid differentiation medium
OGM: organoid growth medium
PARPs: poly (ADP‐ribose) polymerases
PFA: paraformaldehyde
SARS‐CoV‐2: severe acute respiratory syndrome coronavirus 2
TMPRSS2: transmembrane protease serine protease 2
TRAP: telomere repeat amplification protocol

## 1 INTRODUCTION

SARS‐CoV‐2, the coronavirus responsible for the current COVID‐19 pandemic, primarily infiltrates cells through the receptor ACE2 (Hoffmann et al., 2020; Ziegler et al., 2020). This membrane protein is also the receptor of SARS‐CoV which led to an outbreak in 2003 (shi, 2020). Upon binding to ACE2, the SARS‐CoV‐2 spike (S) glycoprotein is mainly cleaved by the type II transmembrane serine proteases (TMPRSS2) at two cleavage sites on separate loops, which primes the S protein for cell entry (Heurich et al., 2014). ACE2 expresses in lung, intestinal and kidney tissues, and thus mediates relative symptoms of COVID‐19 patients, including cough, shortness of breath, fever, fatigue, and gastrointestinal symptoms such as nausea, vomiting, and diarrhea (Mehta et al., 2020). An analysis based on over 700 lung transcriptome samples from severe COVID‐19 patients that present comorbidities revealed that ACE2 is highly expressed in the lungs compared to control individuals (Pinto et al., 2020), suggesting that ACE2 expression level correlates with the severity of COVID‐19.

Like SARS‐CoV, SARS‐CoV‐2 disproportionately affects older individuals, who are more likely to develop severe symptoms and experience higher mortality (O'Driscoll et al., 2020; Williamson et al., 2020). According to the data of The Centers for Disease Control and Prevention, COVID‐19 hospitalization rates are 5–10 times higher in aged people (above 65 years) than in 18–29 years old people, and death rates are 65–340 times higher in aged people (CDC, 2022). Many possible reasons underlie these age‐associated differences, including different cell susceptibility to viruses, existence of underlying chronic diseases, and different immune response and capacity against viral infection (Bartleson et al., 2021; Lewis et al., 2021; Wang, Chiou, et al., 2020; Wang, Chen, et al., 2020). Higher levels of ACE2 have been detected in older human and mice tissues compared to young tissues (Patel & Verma, 2020; Yee et al., 2020).

An inherent aspect of the aging process is the accumulation of DNA damage over time, which can be detected by γH2AX staining (Lopez‐Otin et al., 2013; Mah et al., 2010; Schumacher et al., 2021; Sedelnikova et al., 2004). It has been shown that aging is associated with significant increases in DNA damage found in many different tissues of aged flies, mice, and humans (Moskalev et al., 2013; Soares et al., 2014). Although most DNA lesions arising from extrinsic or intrinsic damage are quickly repaired, a very small number of highly toxic lesions can persist and accumulate, especially DNA damage occurring at telomeres, a distinctive structure located at the ends of chromosomes (Fumagalli et al., 2012; Hewitt et al., 2012). Telomeres consist of hundreds to thousands of TTAGGG tandem repeats and protein complexes known as shelterin. During aging, telomeres progressively shorten and/or accumulate damage caused by cell division or genotoxic stress (Demanelis et al., 2020). Upon DNA damage occurs, the DNA damage response (DDR) signaling pathway is activated and orchestrated by the ataxia telangiectasia mutated (ATM) and ataxia telangiectasia and Rad3‐related (ATR) kinases. Amounts of proteins, including CHK1 and CHK2, are phosphorylated by ATM and ATR to regulate cell cycle progression, DNA repair, DNA replication, and other cellular processes (Marechal & Zou, 2013).

The causal role of DNA damage in aging was verified in mice recently. Two mouse models with “clean” DNA double‐strand breaks through expressing a tetracycline‐controlled SacI restriction enzyme or a I‐PpoI restriction enzyme separately displayed symptoms of premature aging (Kim et al., 2016; White et al., 2015; White & Vijg, 2016). Although the direct role of DNA damage in aging has not been evidenced in humans, numerous premature aging diseases, such as Werner syndrome and Bloom syndrome, have detected increased accumulation of DNA damage caused by mutations in genes involved in the maintenance of genomic stability (Burtner & Kennedy, 2010). In this study, we explored the function of DNA damage in SARS‐CoV‐2 infection and tested whether inhibition of DDR or enhancing DNA repair capacity could alleviate viral load and tissue damage of SARS‐CoV‐2 infection.

## 2 MATERIALS AND METHODS

### 2.1 siRNAs, antibodies, and reagents

The siRNAs targeting human genes were obtained from Invitrogen and JTSBIO Co., Ltd. The target sequences were listed in Table S1. Antibodies used in this manuscript were listed in Table S2. Small molecular compound caffeine was obtained from sigma; KU55933, VE‐822, NMN, and MDL‐800 were obtained from Selleck; etoposide and Doxycycline (Dox) were obtained from sigma.

### 2.2 Viruses, cell culture, and transfection

As previously described (Chi et al., 2020), authentic SARS‐CoV‐2 virus was isolated from the lung lavage fluid of an infected patient. Mouse‐adapted SARS‐CoV‐2 virus was isolated and identified by Changchun Veterinary Research Institute (Yan et al., 2022). Cell infection experiments with infectious SARS‐CoV‐2 were performed in the biosafety level 3 (BSL‐3) facility of Beijing Institute of Microbiology and Epidemiology, Academy of Military Medical Sciences, China. Mice infection experiments were performed in the BSL‐3 facility of Changchun Veterinary Research Institute, Chinese Academy of Agricultural Sciences, China. Human embryonic kidney HEK293T cells, human lung adenocarcinoma Calu‐3 cells, A549 cells, human colorectal adenocarcinoma Caco‐2 cells, human osteosarcoma U2OS cells, and African green monkey kidney cell line Vero‐E6 cells were purchased from American Type Culture Collection (ATCC), and have been tested for mycoplasma contamination previously. Cells were routinely cultured in DMEM (Invitrogen) containing 10% FBS (ExCell Bio). To limit growth discrepancy resulted differences of pseudoviral entry, cell culture medium was replaced to DMEM (Invitrogen) containing 1% FBS (ExCell Bio) after infection. PEI and RNAiMax reagents (Invitrogen) were used for plasmid and siRNA transfection according to the manufacturer's instructions.

### 2.3 Packaging, concentration, and titration of lentivirus and pseudotyped lentivirus

HEK293T cells were cultured in growth media (10% FCS in DMEM) supplemented with L‐Glutamine until 70% confluent on a T‐75 cell culture flask. Cells were then transfected with PEI reagent according to the manufacturer's instructions. Briefly, 20 μg plasmids containing PLP1 (5.88 μg), PLP2 (2.8 μg), VSV‐G (3.92 μg) and pCDH‐Myc‐EGFP (7 μg), or pCDH‐Luc (7 μg), or pHHLVX‐EF1α‐Rluc‐puro (7 μg) were mixed with 58.8 μl PEI reagent in 1.5 ml DMEM without FCS and left for 15 minutes at room temperature. For packaging of pseudotyped lentivirus, VSV‐G was substituted by SARS‐CoV S, SARS‐CoV‐2 S, or MERS‐CoV S. The plasmid‐PEI mixture was then added to the cells, and the cell medium was replaced 6 h later. Forty‐eight hours after transfection, the medium was collected, centrifuged at 1000 *g* for 5 min to remove cell debris, filtered through a 0.45 μm filter, and stored at 4°C. Another 15 ml fresh medium were added into the T‐75 cell culture flask and collected 24 h later. After centrifugation and filter, the medium was combined with the medium stored at 4°C and mixed with 5 × lentivirus concentration solution (50% PEG‐8000, 1.5 M Nacl). After incubation at 4°C overnight, the mix were centrifuged at 2000 *g* for 30 min at 4°C. Carefully remove supernatant and resuspend the viral pellet with 500 μl PBS. To remove serum proteins, the concentrated viral particles were centrifuged at 12,000 *g* for 5 min at 4°C and supernatant was aliquot and stored at −80°C until use. Titration of lentivirus was performed by qRT‐PCR measurement of viral RNA using Lenti‐pac HIV qRT‐PCR Titration Kits (GeneCopoeia) kit according to the manufacturer's instructions. Briefly, lentivirus RNA was purified using TRIzol reagent and treated with DNase I to remove lentiviral expression plasmids. cDNAs were reverse transcribed using random primers and subjected to qPCR for detection of WPRE sequence of lentivirus with the following primers: WPRE‐1, 5′‐CCGTTGTCAGGCAACGTG‐3′ and WPRE‐2, 5′‐AGCTGACAGGTGGTGGCAAT‐3′. Plasmid containing WPRE sequence was gradient diluted and included in each PCR run so that the physical titer of lentivirus could be determined relative to the plasmid DNA sample by the standard curve method. Functional titer of lentivirus was obtained approximately by divide physical titer with 100 (Geraerts et al., 2006).

### 2.4 Establishment, maintenance, differentiation, and virus infection of intestinal organoid

**FIGURE 1:**
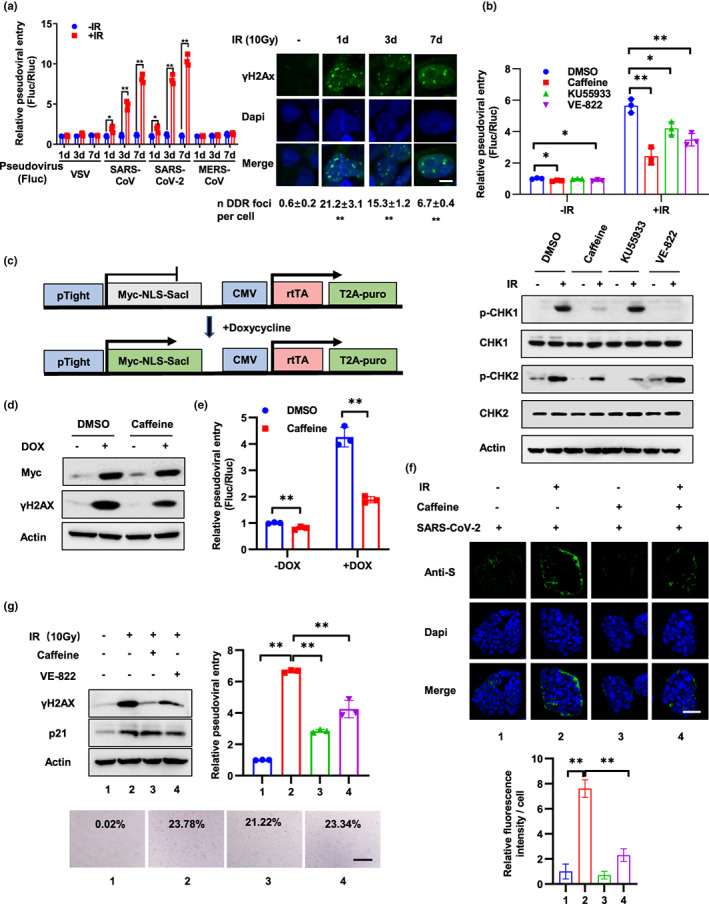
DNA damage promotes SARS‐CoV‐2 entry through DDR. (a) Calu‐3‐Rluc cells (Calu‐3 cells stably expressing Renilla‐luciferase) were treated with 10 Gy IR and followed by infection with lentivirus pseudotyped with the VSV‐G protein or S protein of SARS‐CoV, SARS‐CoV‐2, MERS‐CoV, which harboring a firefly luciferase reporter gene (Fluc) at 1, 3, 7 days after IR. Viral entry was detected 3 days after infection by calculation the ratio of Fluc activity and Rluc activity. DNA damage was detected by immunostaining with an anti‐γH2AX antibody at 1, 3, and 7 days after IR. Scale bar, 10 μm. For the quantification shown, γH2AX foci in around 100 cells per time were analyzed as mean ± SD and differences between indicated group and no‐IR‐treated group were analyzed. (b) Caco‐2‐Rluc cells were treated with caffeine (1 mM), KU55933 (10 μM) or VE‐822 (100 nM) for 12 h prior to IR (10 Gy) treatment. After 2 days, cells were infected with SARS‐CoV‐2 pseudovirus and relative pseudoviral entry was detected 3 days later by luciferase activity assay. Protein expression was examined by Western blot with indicated antibodies at 2 h after IR. (c) Schematic of the SacI expression construct and doxycycline (DOX) treatment. (d and e) Caco‐2‐Rluc cells stably expressing DOX‐induced SacI were treated with or without DOX (2.5 μM) for 3 days and infected with SARS‐CoV‐2 pseudovirus. Expression of Myc‐SacI and γH2AX was detected at 3 days after DOX treatment (d). Relative pseudoviral entry and protein expression were examined at 3 days after infection (e). (f) Caco‐2 cells were treated with caffeine (1 mM) for 12 h prior to IR (10 Gy). Three days later, the cells were incubated with authentic SARS‐CoV‐2 virus for 6 h and the relative virus levels were then detected by immunostaining of SARS‐CoV‐2 S protein. Scale bar, 50 μm. Relative fluorescence intensity per cell was quantified by dividing fluorescence intensity by cell number from >5 representative images and is presented as the mean ± SD. (g) VERO‐E6 cells were treated with IR (10 Gy) and followed by caffeine (1 mM) or VE‐822 (100 nM) at 3 days later. Two days after caffeine or VE‐822 treatment, cells were infected with SARS‐CoV‐2 pseudovirus and relative pseudoviral entry was detected 3 days later by luciferase activity assay. Protein expression and cell senescence were detected by Western blot and SA‐β‐gal staining at 2 days after caffeine or VE‐822 treatment. SA‐β‐gal data were quantified from >5 independent cell counts up to a total of at least 200 cells and are presented as the mean percentage of positive cells. Scale bar, 100 μm. Data are representative of three independent experiments (a–e, g) or two independent experiments (f) (mean ± SD of three [a, b, e, g] biological replicates. *p < 0.05, **p < 0.01)

For organoid establishment, healthy intestinal tissues obtained from Chinese PLA General Hospital were isolated from patients of stomach cancers or necrotizing enterocolitis. The ethics of the study was approved by the Institutional Review Board of Chinese PLA General Hospital (the reference number is S2020‐013‐01). Organoid termed line 0 was isolated from a 41‐day‐old male neonate with necrotizing enterocolitis; organoid termed line 46 was isolated from a 55‐year‐old woman with stomach cancer. The establishment, maintenance, and differentiation of intestinal organoid were performed mainly according to the standard protocol established by Hans Clevers in 2011 (Sato et al., 2011). Briefly, the intestinal tissues were washed and chopped into small pieces. After incubated with Gentle Cell Dissociation Reagent (GCDR, STEMCELL), the tissue sections were pipetted up and down in DMEM with BSA to remove crypts from tissue. Isolated crypts were counted, mixed with Matrigel (Corning), and plated on a 24‐well plate. After the Matrigel dome was polymerized, 300 μl of organoid growth medium (OGM, STEMCELL, components of the medium were illustrated in Table S3) was added and replaced every 2 days. Passage the organoid every 7–10 days by dissolve Matrigel dome with CRS (Corning), disruption the organoid by pipetting up and down vigorously, and re‐plate the organoid with Matrigel. To differentiate organoid, the expansion medium was replaced with organoid differentiation medium (ODM, STEMCELL, components of the medium were illustrated in Table S3) and cultured for 5–8 days. To detect the effect of IR and caffeine on SRAS‐CoV‐2 pseudovirus infection (Figure 1d), organoids were cultured in ODM for 2 days and then treated with IR (10 Gy) or caffeine (1 mM). At 24 h post‐IR, organoids were flipped “inside‐out” in suspension culture with ODM for 2 days to take the apical side of the cells at the outside of the spherical organoid (Co et al., 2019) and then incubated with SARS‐CoV‐2 pseudovirus at approximately MOI = 10 for 3 h and then re‐plated in Matrigel dome. Western blot and immunostaining were performed at 3 days after infection. To detect the effect of MDL‐800 on SRAS‐CoV‐2 pseudovirus infection (Figure 5b,c), organoids were cultured in ODM for differentiation with or without MDL‐800 for 3 days. Then, the organoids were flipped, infected with SARS‐CoV‐2 pseudovirus, and analyzed as above mentioned.

### 2.5 Virus infection in cells

For infection of pseudotyped virus in Caco‐2 and Calu‐3 cells, cultured cells were dissociated with trypsin and cell number was counted. After cells were seeded in a 24‐well plate, pseudotyped virus was added to the cell suspension at MOI = 0.5 and mixed. Virus entry was analyzed at 3 days after virus infection. For infection of authentic SARS‐CoV‐2 virus, adherent Caco‐2 cells were incubated with SARS‐CoV‐2 virus at MOI = 0.1 for 1 h, and then, culture medium was replaced with fresh medium, and virus entry was analyzed at 6 h after virus infection.

### 2.6 Immunofluorescence of cells and organoids

Cell immunofluorescence was performed as previously described (Cheng et al., 2019). Briefly, cells grown on glass coverslips were fixed with 4% PFA (paraformaldehyde), permeabilized with permeabilizing solution (0.5% Triton‐X100, 1% normal goat serum in PBS) and blocked in blocking solution (1% normal goat serum in PBS). The coverslips were then incubated with primary antibodies diluted with blocking solution. Primary antibodies used were illustrated in Table S2. After washed in blocking solution for 3 times, coverslips were incubated with corresponding secondary antibodies. After washed in PBS for 3 times, nuclei were counterstained with 4′,6‐diamidino‐2‐phenylindole (DAPI). For in combination with Tel‐Fish, cells were subjected to immunofluorescence staining and followed by Fish staining of telomere. Briefly, cells after immunofluorescence staining were treated by 4% PFA fixing and PBS washing and incubated with Alexa fluor 488‐OO‐(CCCTAAA)_3_ PNA probe (catalog no. PNA1006, Applied Biosystems) for telomeric DNA hybridization according to the manufacturer's protocol. To detect protein expression and distribution in intestinal organoid, organoid was fixed with 4% PFA for 30 min, washed 3 times with PBS, and dehydration with 20% sucrose in PBS overnight. Organoids were then flash frozen in optimal cutting temperature (OCT) and were cut to 10 μm thick sections using a cryotome. Then, the slides were washed in PBS to remove OCT and permeabilized. The following procedures were the same as cell immunofluorescence. Confocal images were collected using a LSM880 confocal microscope (Zeiss).

### 2.7 qRT‐PCR detection of gene expression and authentic SARS‐CoV‐2 entry

Total cell RNA was extracted using TRIzol reagent according to the manufacturer's instructions (Invitrogen). To detect relative levels of authentic SARS‐CoV‐2 virus, cells infected with SARS‐CoV‐2 were performed with viral RNA extraction with QIAamp Viral RNA Mini Kit (52906, Qiagen) according to the manufacturer's instructions. First‐strand cDNA was reverse transcribed using PrimeScript RT reagent Kit with gDNA Eraser (Takara) and applied for qPCR amplification using TB Green® Premix Ex Taq™ II (Takara) with the following primers: human ACE2 forward, 5′‐AGACCAAAGCATCAAAGTGAGGAT‐3′; human ACE2 reverse, 5′‐TTAAAGGAGATTCTTGGTTTCAAATTAGCC‐3′; human β‐actin forward, 5′‐AGAAGAGCTACGAGCTGCCTGA‐3′; human β‐actin reverse, 5′‐CAATGATCTTGATCTTCATTGTGCT‐3′; human DPP4 forward, 5′‐AAGTGGCGTGTTCAAGTGTG‐3′; human DPP4 reverse, 5′‐ATGGTCAAGGTTGTCTTCTGG‐3′; human IFN‐beta forward, 5′‐AATTGAATGGGAGGCTTGAATACTGCCTCAAGG‐3′; human IFN‐beta reverse, 5′‐GTCTCATTCCAGCCAGTGCTAGATGAATCTTGT‐3′; human ISG15 forward, 5′‐ATGTCGGTGTCAGAGCTGAAGGCG‐3′; human ISG15 reverse, 5′‐CTTGTTATTCCTCACCAGGATGCTCAGAGGTT‐3′; human OAS1 forward, 5′‐CAGCAACTCTGCATCTACTGGAC‐3′; human OAS1 reverse, 5′‐TCAGCCTCTTGTGCCAGCTGC‐3′. SARS‐CoV‐2 N forward, 5′‐GGGGAACTTCTCCTGCTAGAAT‐3′; SARS‐CoV‐2 N reverse, 5′‐CAGACATTTTGCTCTCAAGCTG‐3′. The relative expression was calculated by the comparative Ct method.

### 2.8 TERC knockout Calu‐3 cells

TERC knockout Calu‐3 cells were generated by CRISPR‐Cas9. Calu‐3‐Cas9 stable cells were constructed by transfection with lentivirus expressing Cas9, which was packaged in 293T cells by co‐expression of lentiCas9‐Blast (Addgene #52962), psPAX2 (Addgene #12260) and pCMV‐VSV‐G (Addgene #8454). The sgRNA (single guide RNA) sequence was cloned into the lentiGuide‐Puro (Addgene #52963) and packaged into lentivirus in 293T cells, followed by transfection into Calu‐3‐Cas9 cells. Two sgRNAs targeting TERC were used, sgTERC‐pro targets a region located in promoter of TERC, and sgTERC‐NAR targets a coding region of TERC which was reported in a research published on Nucleic Acids Research (Min et al., 2017). sgRNA sequences were listed in Table S4. After selection with puromycin, monoclonal cells were selected, cultured for approximately 30 passages, and collected for genomic DNA extraction and amplification by PCR with the following primers: Forward, 5′‐GTCCTTAAGATTAATAATGTAGTAGTTACACTTGATTAAAGCCA‐3′; Reverse, 5′‐GCATGTGTGAGCCGAGTCCT‐3′. The PCR products were sequenced.

### 2.9 Mouse challenge experiment

Young (8‐week) and older (12‐month) C57BL/6J female mice were obtained from Vital River. All experiments were carried out in accordance with the Guide for the Care and Use of Laboratory Animals published by the United States National Institutes of Health after securing the approval of the Committee of Animal Care of the Beijing Institute of Biotechnology. For intranasal infection, mice were anesthetized with sodium pentobarbital at a dose of 50 mg/kg though intraperitoneal route and then intranasally infected with 4.3 × 10^5^ pfu of SARS‐CoV‐2. All mice were sacrificed on Day 3 post‐infection for lung tissue processing.

### 2.10 Histopathological analysis

Mouse tissues were excised and fixed with 10% neutral buffered formalin, dehydrated, and embedded in paraffin. Each embedded tissue was sectioned into 4 μm thickness longitudinal sections. Three tissue sections derived from different parts of each tissue were stained with hematoxylin and eosin (H&E) according to standard procedures for examination by light microscopy. The degree of lung damage under the light microscopy was assessed by the degeneration of alveolar epithelial cells, the expansion of parenchymal wall, edema, hemorrhage, and inflammatory cells infiltration. For the degree of thickened alveolar septa, we scored 0 when no alveolar septa were thickened, scored 1 when the thickened alveolar septa was less than 10%, and scored 2 when the thickened alveolar septa was above 10%. For the degree of alveolar damage, we scored 0 when no alveolar epithelial cell damage was observed, scored 1 when the alveolar epithelial cell damage was less than 10%, and scored 2 when the damage was 10%–50%. For the degree fibrin exudation, we scored 0 when no fibrin exudation was detected, scored 1 when the area of the fibrin exudation was less than 10%, and scored 2 when the area was 10%–50%. For the degree of vascular congestion, we scored 0 when no vascular congestion was observed and scored 1 when area of vascular congestion was less than 10%. For the degree of inflammatory cells infiltration, we scored 0 when no inflammatory cell infiltration, scored 1 when occasional infiltration of single inflammatory cell was visible, and scored 2 when focal infiltration of inflammatory cells was visible. The scores of these parameters were cumulated to provide a total score per animal to assess the severity of tissue damage. The total HE scoring criteria were as follows: 0 (total score = 0); 1 (total score = 1–2); 2 (total score = 3–4); 3 (total score = 5–6); and 4 (total score = 7–9).

### 2.11 Clinical samples and immunohistochemistry

Healthy intestinal tissues obtained from Chinese PLA General Hospital were isolated from patients of stomach cancers or right‐sided colon cancers. The ethics of the study was reviewed and approved by the Institutional Review Board of Chinese PLA General Hospital (the reference number is S2020‐013‐01). Immunohistochemical staining was performed as described previously (Cheng et al., 2019). Briefly, the formalin‐fixed paraffin sections were deparaffinized, rehydrated, and pre‐treated with 3% H_2_O_2_ for 25 min. The antibody‐binding epitopes of the antigens were retrieved by microwave treatment, and the sections were then preincubated with 3% BSA to block nonspecific binding. The slides were then incubated with primary antibodies and subsequent secondary antibodies. The resource and dilution of primary antibodies used in the study were listed in Table S2. Each specimen was assigned a score generated by multiplying the intensity of the staining (no staining = 0, weak staining = 1, moderate staining = 2, and strong staining = 3) by the percentage of stained cells (0%–100%). The optimal cutoff value of the IHC scores was estimated using receiver operating characteristic (ROC) curve analysis.

### 2.12 Statistical analysis

Statistical significance in cell susceptibility to pseudotyped virus or authentic virus, viral loads, gene expression levels, and gene enrichment in ChIP assay were assessed by multiple *t*‐tests. The correlation of ACE2, p‐c‐Jun, and γH2Ax was determined using Pearson correlation coefficient. All statistical tests were two‐sided. Statistical calculations were performed using PRISM9. In all assays, *p* < 0.05 was considered statistically significant.

## 3 RESULTS

### 3.1 DNA damage promotes SARS‐CoV‐2 entry through DDR

Pseudovirus system is well suited for virus entry assays as they allow viral entry to be distinguished from other virus life cycle steps, such as viral replication, packaging, and release and has been widely used in research of molecular mechanisms of SARS‐CoV‐2 entry (Cantuti‐Castelvetri et al., 2020; Daly et al., 2020). To detect whether DNA damage regulates SARS‐CoV‐2 virus entry, we exposed Calu‐3‐Rluc cells (Calu‐3 cells stably expressing Renilla‐luciferase) to 10 Gy of gamma ionizing radiation (IR) to induce DNA damage. At different days after IR, the cells were infected with lentivirus pseudotyped with S protein of SARS‐CoV‐2, SARS‐CoV, MERS‐CoV, or VSV‐G protein. A firefly luciferase (Fluc) gene was introduced into this pseudovirus, and thus, pseudoviral entry was examined by luciferase activity assay at 3 days after viral infection (Figure 1a). To limit cell number discrepancy resulted from IR‐induced cell growth arrest, all cells were cultured in DMEM medium supplemented with 1% FBS after viral infection. Cell grows slow under 1% FBS, and no significant differences were detected between IR‐treated and non‐treated cells (Figure S1a). Notably, cell susceptibility to SARS‐CoV‐2 and SARS‐CoV pseudovirus increased progressively at 1, 3, and 7 days after IR treatment and DNA damage maintained at a high level during this time course. In contrast, cell susceptibility to MERS‐CoV and VSV pseudovirus was not affected by IR treatment (Figure 1a). Similar results were observed in Caco‐2‐Rluc cells (Figure S1b). Lower doses of IR (1 Gy) treatment also promote SARS‐CoV‐2 and SARS‐CoV pseudoviral entry but both viral entries were decreased at 7 days after IR compared to 3 days after IR (Figure S1c), which can be explained by the findings that DNA damage was largely repaired at 7 days after IR because the number of γH2AX foci almost decreased to the basal level (Figure S1c).

In addition to DNA, many other macromolecules in cells could be damaged by IR treatment. To examine the specificity of the role of DNA damage in viral entry, Caco‐2‐Rluc cells were treated with the DDR inhibitor Caffeine, KU55933, or VE‐822. These inhibitors dramatically inhibited the phosphorylation of CHK1 or CHK2 (Figure 1b). Importantly, SARS‐CoV‐2 pseudoviral entry was also inhibited by these agents (Figure 1b). Furthermore, knockdown of ATM and ATR, two critical mediators of the DDR, also reduced viral entry (Figure S1d). These results suggested that IR regulated viral entry mainly through DNA damage and inhibition of DDR could relieve the effect of IR. Additionally, the DNA damage induced by other genotoxic agents, including etoposide and UV, also promoted SARS‐CoV‐2 pseudovirus infection, and the effect was inhibited by Caffeine (Figure S1e,f). Moreover, to provide a direct link between DNA damage and viral entry, we constructed a doxycycline‐induced restriction endonuclease SacI system to generate a clean DNA double‐strand break (DSB) without other lesions (Figure 1c; Maslov et al., 2009). After SacI was activated by DOX in Caco‐2‐Rluc cells, expression of γH2AX was significantly increased (Figure 1d). Notably, pseudoviral entry was increased dramatically and Caffeine inhibited the effect (Figure 1e). These results support the idea that DNA damage and subsequent DDR promote SARS‐CoV‐2 pseudoviral entry.

We next explored the role of DNA damage and DDR in cell susceptibility to authentic SARS‐CoV‐2 virus infection. Caco‐2 cells were treated with Caffeine for 12 h prior to IR and incubated with authentic SARS‐CoV‐2 virus for 6 h, and the relative virus levels were then detected by immunostaining of SARS‐CoV‐2 S protein (Figure 1f) or RNA levels of N (Figure S1g). In agreement with the results of pseudoviral entry, authentic viral entry was enhanced by IR treatment and inhibited by Caffeine. Since intestinal organoids have been validated as a promising model of SARS‐CoV‐2 infection (Lamers et al., 2020; Zhou, Li, et al., 2020; Zhou, Yang, et al., 2020), we isolated intestinal stem cells from a patient with stomach cancer (55‐year‐old woman, Line 46) and a necrotizing enterocolitis (neonate, Line 0) and cultured them as organoids. Then, organoids were treated with IR or Caffeine, flipped “inside‐out” in suspension culture, and then incubated with SARS‐CoV‐2 pseudovirus. Viral infection efficiency and DNA damage were detected at 3 days after infection. Consistent with the results in cells, the susceptibility of the organoids to SARS‐CoV‐2 pseudovirus was enhanced by IR treatment and inhibited by Caffeine treatment (Figure S1h). Therefore, these results indicate that DNA damage facilitates SARS‐CoV‐2 infection and the effect is restrained by inhibition of DDR.

Next, we sought to explore the function of DNA damage in SARS‐CoV‐2 infection in VERO‐E6 cells, a normal kidney cell line isolated from Cercopithecus aethiops. Ten Gy of IR treatment induced DNA damage, p21 upregulation and cell senescence in VERO‐E6 cells and importantly, enhanced SARS‐CoV‐2 pseudoviral entry (Figure 1g). DDR inhibitor Caffeine and VE‐822 treatment at 3 days after IR reduced DNA damage but did not prevent cells from senescence. Notably, Caffeine and VE‐822 inhibited SARS‐CoV‐2 psuedoviral entry, which addressed the role of DNA damage in SARS‐CoV‐2 infection in senescent cells (Figure 1g). Moreover, to test whether senescent cells with DNA damage are more susceptible to viral entry compared to senescent cells without DNA damage, we overexpressed p16 in VERO‐E6 cells to induce cell senescence without DNA damage. Notably, these cells did not show elevated susceptibility to SARS‐CoV‐2 pseudoviral entry (Figure S1i). Collectively, these data highlight the role of DNA damage and DDR in SARS‐CoV‐2 infection.

### 3.2 DNA damage enhances ACE2 expression by activation of c‐Jun

**FIGURE 2:**
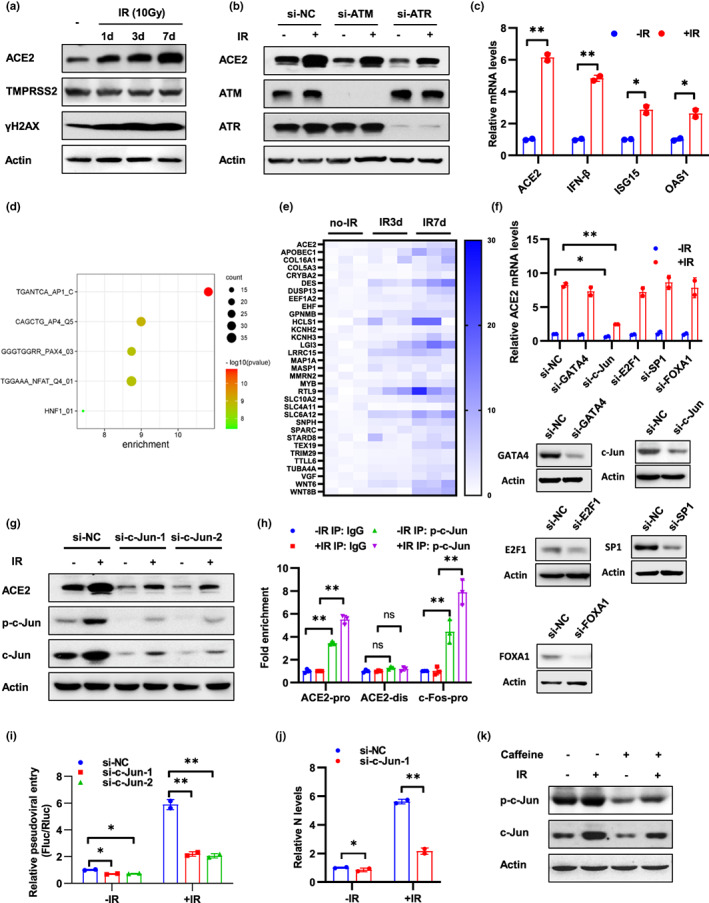
DNA damage enhances ACE2 expression by activation of c‐Jun. (a) Calu‐3‐Rluc cells at 1, 3, and 7 days after 10 Gy IR treatment were subjected to Western blot analysis using the indicated antibodies. (b) Caco‐2 cells transfected with siRNA of ATM or ATR were treated with 10 Gy IR and followed by Western blot analysis using the indicated antibodies at 3 days after IR. (c) Calu‐3‐Rluc cells at 3 days after IR treatment (10 Gy) were subjected to qRT‐PCR analysis of mRNA levels of indicated genes. (d) Calu‐3‐Rluc cells after IR treatment were analyzed by RNA‐seq, and top 150 up‐regulated genes 3 days after IR were performed with TFT (all transcription factor targets) analysis. (e) Expression of ACE2 and 32 targets of AP1 signaling was illustrated. (f) Caco‐2 cells transfected with indicated siRNA were treated with 10 Gy IR and ACE2 mRNA levels were detected at 48 h after IR. The effect of knockdown was detected by Western blot analysis of target genes. (g) Caco‐2 cells transfected with siRNAs of c‐Jun were treated with 10 Gy IR and followed by Western blot analysis using the indicated antibodies at 3 days after IR. (h) ChIP analysis of the occupancy of p‐c‐Jun on the promoter region (ACE2 pro) and distal region (ACE2 dis) of ACE2 in Caco‐2 cells at 3 h after 10 Gy IR treatment. The c‐Fos promoter (c‐Fos pro) containing an AP‐1 binding site was included as a positive control. IgG, normal serum. (i and j) Caco‐2‐Rluc cells transfected with c‐Jun siRNAs were treated with 10 Gy IR and subjected to SARS‐CoV‐2 pseudovirus infection (i) or SARS‐CoV‐2 authentic virus infection (j) at 3 days after IR. Viral entry was analyzed by luciferase activity at 3 days after infection (i) or by qRT‐PCR analysis of SARS‐CoV‐2 N expression at 6 h after infection (j). (k) Caco‐2 cells were pre‐treated with caffeine (1 mM) for 12 h and then subjected to IR (10 Gy) treatment. Western blot analysis was performed using the indicated antibodies at 3 days after IR. Data are representative of three independent experiments (a–c, f–i, k) or two independent experiments (j) (mean ± SD of three (h) or two biological replicates (c, f, i, j). *p < 0.05, **p < 0.01)

Because the entry of SARS‐CoV‐2 and SARS‐CoV, but not VSV and MERS‐CoV, was enhanced by DNA damage, we hypothesize that DNA damage and DDR may promote viral entry through regulation of ACE2, the receptor of both SARS‐CoV‐2 and SARS‐CoV. Firstly, we detect whether DNA damage regulates the expression of ACE2. As anticipated, IR treatment markedly increased the expression of ACE2, but not TMPRSS2, another key protein involved in SARS‐CoV‐2 entry, or DPP4, the receptor of MERS‐CoV (Figure 2a and Figure S2a,b). Moreover, ACE2 expression was induced by other genotoxic agents and impaired after inhibiting the DDR pathways by treatment with si‐ATM, si‐ATR, or Caffeine (Figure 2b and Figure S2c–e). These observations suggested that DNA damage and subsequent DDR upregulate ACE2 expression, which was consistent with the findings of viral entry. We also detected ACE2 expression in IR‐induced senescent cells after a long‐term culture. Expression of ACE2 and pseudoviral entry was enhanced in cells which cultured for 2 weeks after IR (Figure S2f).

Next, we aimed to detect the mechanism of regulation of DNA damage on ACE2 expression. IR up‐regulated mRNA levels of ACE2 in Calu‐3‐Rluc cells and inhibition of DDR by Caffeine, KU55933, or VE‐822 alleviates IR‐induced ACE2 expression (Figure 2c and Figure S3a). Reciprocally, DNA damage did not regulate ectopic ACE2 expression in A549 cells which lacks endogenous ACE2 protein (Figure S3b), suggesting that DNA damage regulate ACE2 expression at the transcriptional level. Moreover, DNA damage did not influence pseudoviral entry in A549‐ACE2 cells (Figure S3b), which indicates that upregulation of ACE2 expression is responsible for DNA damage‐induced viral entry. To exclude other possible mechanisms involved in regulation of viral entry by DNA damage, pseudoviral entry was examined in Caco‐2 cells transfected with ACE2 siRNA. Knockdown of ACE2 abolished IR‐induced viral entry, which highlight the role of ACE2 in DNA damage‐mediated viral entry (Figure S3c). IFN‐β was reported to increase ACE2 mRNA level through the transcription factor Stat1 (Ziegler et al., 2020), and IR treatment increased the expression of IFN‐β and its target genes ISG15 and OAS1 (Figure 2c). We next sought to validate the role of IFN‐β and Stat1 in DNA damage‐induced ACE2 expression. Knockdown of Stat1 did not regulate ACE2 protein levels in the presence or absence of IR (Figure S3d), which could be explained by a recent study showing that interferons stimulated the expression of a truncated form of ACE2, which could not mediate viral entry (Onabajo et al., 2020). To obtain a global view of the signaling pathways that could contribute to ACE2 transcription after IR treatment, Calu‐3‐Rluc cells after IR treatment were analyzed by RNA‐seq. Differential expressed genes and related analysis were provided in Table S5. Expression of ACE2 increased in IR‐treated cells (Figure 2e). To analyze transcription factors response to IR, top 150 up‐regulated genes after IR were performed with TFT (all transcription factor targets) analysis on GSEA website. The most enriched transcription signaling pathway was AP1 pathway in which 32 target genes were up‐regulated (Figure 2d,e). AP1 pathway is mainly mediated by transcription factor c‐Jun and c‐Fos. Additionally, c‐Jun was predicted to bind to ACE2 promoter according to the JASPR database. Next, we detected ACE2 expression in cells transfected with c‐Jun and other predicted transcription factors (Figure 2f). Knockdown of c‐Jun, but not other transcription factors, significantly inhibited ACE2 expression (Figure 2f,g). To address whether c‐Jun binds to the promoter of ACE2, we performed a ChIP assay and verified that phosphorylated c‐Jun (p‐c‐Jun) bound to the ACE2 promoter region and a reported promoter region of c‐Fos (Hayakawa et al., 2004), but not to a distal region of ACE2 (Figure 2h).

Next, we examined the role of c‐Jun in DNA damage‐induced viral entry. Knockdown of c‐Jun inhibited cell susceptibility to SARS‐CoV‐2 pseudovirus and authentic virus (Figure 2i,j), which suggested that c‐Jun is required for DNA damage‐induced viral entry. Moreover, both total and phosphorylated c‐Jun levels were upregulated by IR treatment and inhibited by Caffeine (Figure 2k and Figure S3e). Finally, we examined the regulatory mechanism in human intestinal organoids. Consistently, IR treatment promoted the expression of ACE2 and p‐c‐Jun in neonate organoid (Line 0) and inhibition of DDR by Caffeine reduced ACE2 and p‐c‐Jun expression in aged organoid (Line 46; Figure S3f). Collectively, these data indicate that DNA damage promotes SARS‐CoV‐2 infection through upregulation of ACE2 expression and c‐Jun is required for this process.

### 3.3 Telomere attrition or uncapping promote SARS‐CoV‐2 entry

**FIGURE 3:**
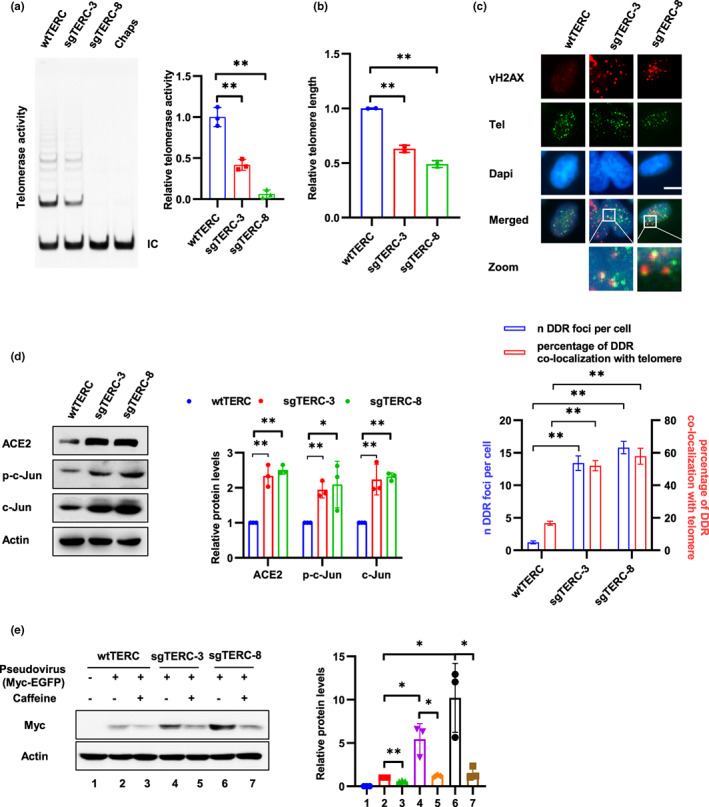
Telomere attrition promotes SARS‐CoV‐2 entry. (a) Telomerase activities of Calu‐3 sgTERC single clones (sgTERC‐3 and sgTERC‐8) were detected using TRAP assay. Chaps buffer was used as a negative control for TRAP assay. IC, internal control. Relative telomerase activity was calculated as the ratio of the intensity of the telomerase ladder over the intensity of the IC. (b) Relative telomere length was examined in sgTERC‐3 and sgTERC‐8 cells using qPCR. (c) Telomere damage was detected by immunostaining with an anti‐γH2AX antibody, followed by Tel‐Fish to detect telomeres. Scale bar, 10 μm. Telomere damage was quantified from at least 200 cells and are presented as the mean ± SD. (d) sgTERC‐3 and sgTERC‐8 cells were subjected to Western blot analysis using indicated antibodies. (e) Calu‐3 sgTERC single clones (sgTERC‐3 and sgTERC‐8) and wild‐type Calu‐3 cells (wtTERC) were treated with caffeine (1 mM) for 12 h and infected with SARS‐CoV‐2 pseudovirus harboring Myc‐EGFP. Viral entry was analyzed by Western blot at 3 days after infection using anti‐Myc antibody. Data are representative of three independent experiments (a–e) (mean ± SD of three (a) or two biological replicates (b) or three independent experiments (d and e). *p < 0.05, **p < 0.01)

Telomere length decreases progressively with cell division and telomerase compensates telomere repeats during each cell cycle in stem cells and most cancer cells to maintain telomere length (Shay, 2016). To address whether telomere attrition contribute to cell susceptibility to SARS‐CoV‐2, we first generated Calu‐3‐sgTERC cells by CRISPR‐Cas9 mediated knockout of TERC gene which encode an RNA template for telomere replication by telomerase. To improve inhibition efficiency, two sgRNAs of TERC (sgTERC‐pro, sgRNA sequence located at the promoter region of TERC gene, sgTERC‐NAR, a previous reported sgRNA which located at the coding region of TERC gene [Min et al., 2017]) were introduced into cells. Single clones were screened by DNA sequencing and telomere repeat amplification protocol (TRAP) to verify the deletion or insertion of the TERC locus and the loss of telomerase activity as reported (Min et al., 2017). Both alleles of TERC locus were edited in two single clones (sgTERC‐3 and sgTERC‐8; Figure S4a). Notably, compared to wild‐type (wt) cells, single clones displayed reduced telomerase activity (Figure 3a). The cells were cultured for approximately 30–35 passages from single cell to the amount enough for identification and viral infection assay. Mean telomere length was decreased and telomere damage was increased in sgTERC cells (Figure 3b,c) Additionally, cell cycle arrested at the G2/M phase in sgTERC cells (Figure S4b) as reported (Jullien et al., 2013).

Telomere damage is considered as a irreparable lesion and causes persistent DDR activation (Fumagalli et al., 2012). Consistently, expression of ACE2 and c‐Jun was upregulated in these TERC knockout cells and caffeine inhibited ACE2 expression (Figure 3d and Figure S4c). Next, these cells were infected with SARS‐CoV‐2 pseudovirus containing a Myc‐EGFP gene, and then, viral entry can be measured via detection the expression of Myc‐EGFP using Western blot with Myc antibody, because EGFP signal was too weak to observe by fluorescence microscopy. Notably, sgTERC‐3 and sgTERC‐8 cells were more susceptible to SARS‐CoV‐2 pseudovirus infection than wild‐type Calu‐3 cells and Caffeine treatment inhibited the effect (Figure 3e). Moreover, TPP1ΔRD and POT1ΔOB, two mutants of shelterin complex which is essential for protecting telomere ends, were transiently transfected into Caco‐2 cells to induce telomere uncapping and telomere damage (Figure S5a; Rai & Chang, 2017). Expression of ACE2 and c‐Jun was increased in cells transfected with the mutants and Caffeine inhibited increasing of ACE2 (Figure S5b,c). Consistently, SARS‐CoV‐2 pseudoviral infection was elevated in telomere uncapping cells, and the effect was inhibited by Caffeine (Figure S5d). These results suggest that DNA damage at telomeres, either caused by telomere attrition or telomere uncapping, upregulates expression of ACE2 and promotes SARS‐CoV‐2 entry.

### 3.4 DNA damage and DDR increase viral loads and lung damage in mice

**FIGURE 4:**
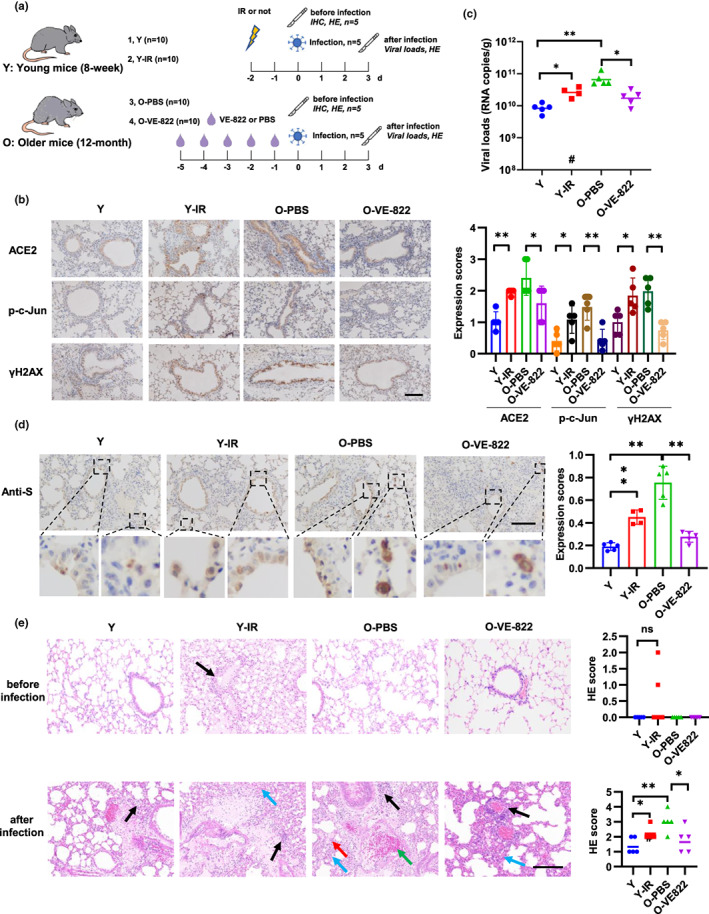
DNA damage and DDR increase viral loads and lung damage in mice. (a) Schematic of mice treatment and viral infection. Young C57BL/6J mice (8‐week‐old) were treated with 15 Gy of IR, and older mice (12‐month‐old) were treated with VE‐822 (oral administration, 60 mg/kg/day). The mice were then sacrificed for immunohistochemistry staining (*n* = 5) (b) or intranasally infected with 4.3 × 10^5^ PFU of SARS‐CoV‐2 mouse‐adapted virus and sacrificed on 3 dpi for tissue collection (*n* = 5) (c–e). (b) Expression of ACE2, p‐c‐Jun, and γH2AX in lung sections of mouse. Representative immunohistochemical staining was illustrated (left). Scale bar, 100 μm. Symbols represent data from individual mouse, and bars represent the mean ± SD of protein expression (right). (c) SARS‐CoV‐2 genomic RNA copies in mouse lung homogenates were determined by qRT‐PCR. Symbols represent data from individual mouse, and bars represent the geometric means of viral titers. #, one mouse died after anesthetization. (d) Immunohistochemistry staining of SARS‐CoV‐2 S protein in mouse lung sections. The dash box is magnified at the bottom of the same image. Scale bar, 100 μm. (e) H&E staining of mouse lung sections. Arrows indicate multifocal lesions with inflammatory infiltration (black), alveolar septal thickening (blue), fibrin exudation (red), and vascular congestion (green). Scale bar, 100 μm. Data from semiquantitative analysis of histopathological changes of lung tissues (HE scores) are presented as geometric means. #, one mouse died after anesthetization. *p < 0.05, **p < 0.01

To investigate the function of DNA damage in vivo, we treated young mice (8‐week, Y) with IR and older mice (12‐month, O) with VE‐822, which inhibit SARS‐CoV‐2 psuedoviral entry in vitro (Figure 1b). VE‐822 is a selective ATR inhibitor and inhibits CHK1 phosphorylation and subsequent DDR pathway. The safety of VE‐822 has been proved in a phase 2 clinical trial to test the anti‐tumor activity of VE‐822 combined with gemcitabine in high‐grade serous ovarian cancer (Konstantinopoulos et al., 2020). Forty mice were divided into four groups (Y, Y‐IR, O‐PBS, and O‐VE‐822, *n* = 10 each group). At 2 days after IR or 5 days after VE‐822 treatment, 5 mice of each group were sacrificed and performed with IHC and HE staining before SARS‐CoV‐2 infection. Another 5 mice of each group were then intranasally infected with a mouse‐adapted SARS‐CoV‐2 variant C57MA14 (Yan et al., 2022) and sacrificed for detection of viral loads and lung damage (Figure 4a). ACE2 expression was detected in bronchial epithelial cells and part of alveolar epithelial cells but not in perivascular regions (Figure S6a), which is consisted with a recent published data based on scRNA‐seq and immunofluorescence assay (Muhl et al., 2022). p‐c‐jun and gammaH2AX were detected in all cell types (Figure S6a). Consisted with the results of in vitro experiments, IR treatment increased the expression of ACE2 and p‐c‐Jun expression in bronchial epithelial cells and alveolar epithelial cells in young mice (Figure 4b and Figure S6b). Importantly, expression of ACE2, p‐c‐Jun, and γH2AX was stronger in older mice than in young mice, and their expression was inhibited by VE‐822 treatment (Figure 4b and Figure S6b). Then, we detected viral loads in lung tissue of mice inoculated with SARS‐CoV‐2 virus at 3 days post‐infection (dpi). IR treatment increased the levels of viral RNA and S protein expression in lung tissues of young mice (Figure 4c,d). Compared to young mice, older mice had a higher level of viral loads and S protein expression, which decreased after treated with VE‐822 (Figure 4c,d). Moreover, hematoxylin and eosin (H&E) staining of lung tissues indicated pneumonia and tissue damage, characterized with thickened alveolar septa, alveolar damage, fibrin exudation, vascular congestion, and activated inflammatory cell infiltration. Firstly, we detected tissue damage in IR or VE‐822 treated mice before infection and no significant differences was detected compared to control mice although two mice after IR displayed slight lung damage (Figure 4e). In mice after infection, lung damage was milder in young mice than in older mice. IR treatment aggravated lung damage in young mice and VE‐822 alleviated lung damage in older mice (Figure 4e and Figure S6c). These results indicate that DNA damage accumulated during aging increased SARS‐CoV‐2 viral loads and lung damage which can be decreased by inhibition of DNA damage response.

**FIGURE 5:**
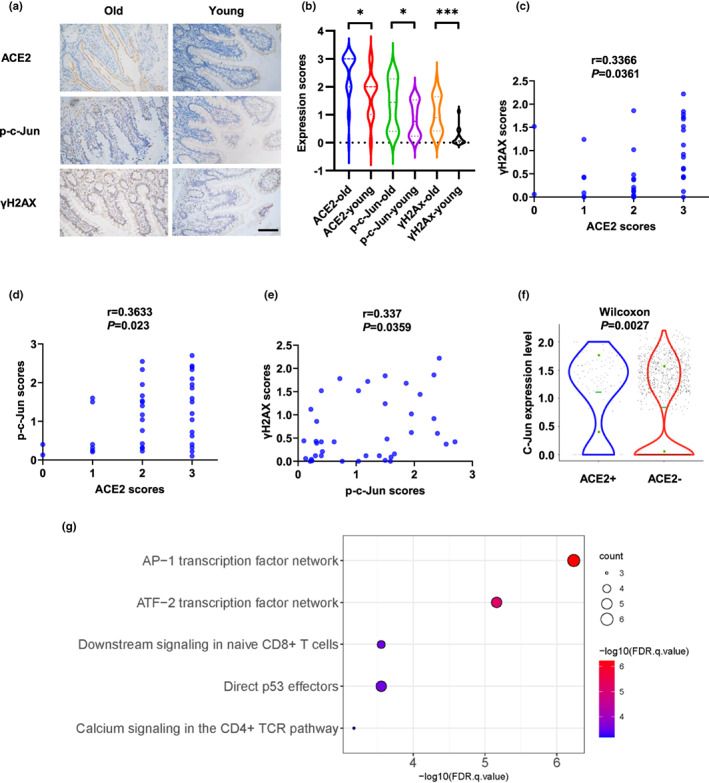
ACE2 correlates positively with p‐c‐Jun and γH2AX in human tissues. (a and b) Expression of ACE2, p‐c‐Jun, and γH2AX in old (60–73 years old, *n* = 24) and young (31–50 years old, *n* = 15) human intestinal tissues. Representative immunohistochemical staining of ACE2, p‐c‐Jun and γH2AX is shown in (a). Scale bar, 100 μm. (c–e) Correlations between expression of ACE2 and γH2AX (c), ACE2 and p‐c‐Jun (d), and p‐c‐Jun and γH2AX (e) were analyzed. (f) Expression of c‐Jun in ACE2+ and ACE2‐ nasal ciliated 2 cells. (g) Pathway Interaction Database (PID) analysis of 73 up‐regulated genes in ACE2+ nasal ciliated 2 cells. *p < 0.05, ***p < 0.001

### 3.5 ACE2 correlates positively with p‐c‐Jun and γH2AX in human tissues

To approach the clinical evidence of the role of DNA damage and DDR in SARS‐CoV‐2 infection during aging, we analyzed the expression of ACE2, γH2AX, and p‐c‐Jun in healthy human small intestinal tissues isolated from old (60–73 years old) and young (31–50 years old) patients with stomach cancers or right‐sided colon cancers. The expression of ACE2, γH2AX, and p‐c‐Jun was higher in small intestine tissues of old individuals (Figure 5a,b). Additionally, ACE2 expression positively correlated with γH2AX and p‐c‐Jun expression in human intestinal tissues (Figure 5c–e). The nasal cavity is typically the primary site of SARS‐CoV‐2 infection and thus plays an important role in virus transmission (Gallo et al., 2020). Thus, we compared the expression of c‐Jun in ACE2^+^ and ACE2^−^ human nasal ciliated 2 cells, which have been identified to express highest level of ACE2 in nasal cavity (Hou et al., 2020), using a published scRNA‐seq data (Vieira Braga et al., 2019). c‐Jun expression was significantly upregulated in ACE2^+^ cells compared with ACE2^−^ cells (Figure 5f). Additionally, a Pathway Interaction Database (PID) analysis of 73 upregulated genes in ACE2^+^ ciliated 2 cells indicated the AP‐1 and ATF2 signaling pathways were the most significantly enriched (Figure 5g, Table S6). Consistently, these pathways could be activated by DNA damage and c‐Jun plays a major role in the signal transduction (Christmann & Kaina, 2013; Karin, 1995). Taken together, these findings provided clinical evidences of the correlation of DNA damage and expression of c‐Jun and ACE2 in human tissues.

### 3.6 NMN treatment alleviates SARS‐CoV‐2 infection and lung damage

**FIGURE 6:**
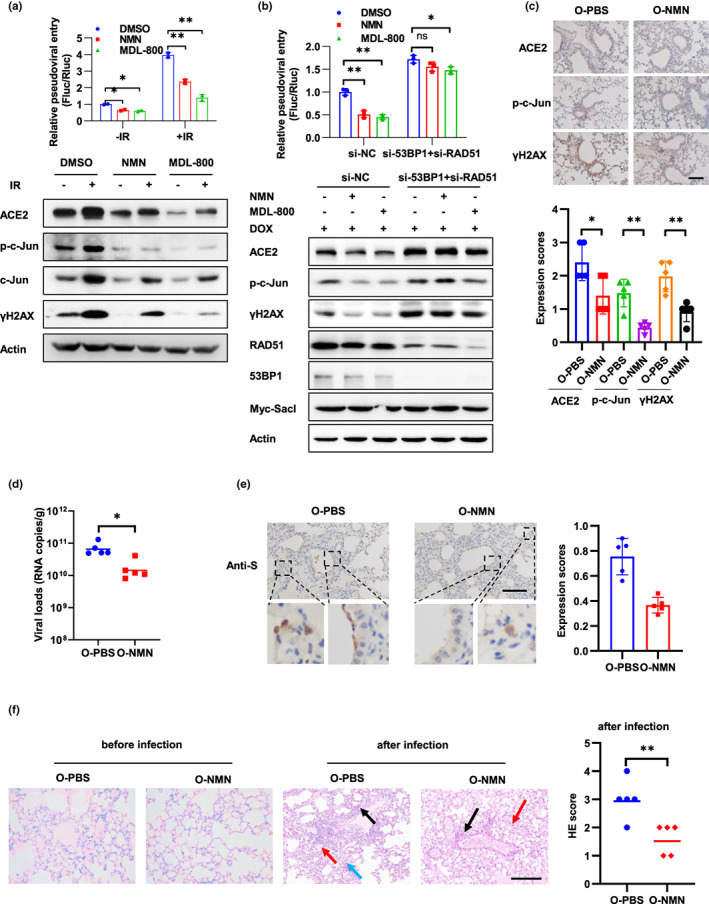
NMN alleviates SARS‐CoV‐2 infection and lung damage. (a) Caco‐2 cells were treated with NMN (1 mM) or MDL‐800 (20 μM) for 7 days and followed by 10 Gy IR. The cells were infected with the SARS‐CoV‐2 pseudovirus at 3 days post‐IR, and viral entry was analyzed by luciferase activity at 3 days after infection. The expression of the indicated proteins was analyzed by Western blot. (b) Caco‐2‐Rluc cells stably expressing DOX‐induced SacI were treated with DMSO, NMN (1 mM), or MDL‐800 (20 μM) for 7 days prior to transfection with 53BP1 and RAD51 siRNAs, followed by DOX (2.5 μM) treatment for 3 days. Then, the cells were harvested for Western blot analysis to detect indicated protein expression or infected with SARS‐CoV‐2 pseudovirus and the relative pseudoviral entry was analyzed at 3 days after infection. (c–f) Older mice (12‐month‐old) were pre‐treated with PBS or NMN (oral administration, 200 mg/kg/day) for 7 days and then sacrificed for immunohistochemistry staining to detect protein expression (c) (*n* = 5 for each group) or intranasally infected with 4.3 × 10^5^ PFU of SARS‐CoV‐2 mouse‐adapted virus and sacrificed on 3 dpi for tissue collection (d–f) (*n* = 5 for each group). (c) Expression of ACE2, p‐c‐Jun, and γH2AX in mouse lung sections was detected, and representative immunohistochemical staining was illustrated. Scale bar, 100 μm. Symbols represent data from individual mouse, and bars represent the mean ± SD of protein expression (right). (d) SARS‐CoV‐2 genomic RNA copies in mouse lung homogenates. Symbols represent data from individual mouse, and bars represent the geometric mean of viral titers. (e) Immunohistochemistry staining of SARS‐CoV‐2 S protein in mouse lung sections. The dash box is magnified at the bottom of the same image. Scale bar, 100 μm. (f) H&E staining of mouse lung sections. Arrows indicate multifocal lesions with inflammatory infiltration (black), alveolar septal thickening (blue), and fibrin exudation (red). Scale bar, 100 μm. Data from semiquantitative analysis of histopathological changes of lung tissues are presented as geometric means. Data are representative of three independent experiments (a, b) (mean ± SD of two (a) or three biological replicates (b), *p < 0.05, **p < 0.01)

The decline of DNA repair capacity with age strongly contributes to the age‐associated accumulation of DNA damage (Li & Vijg, 2012). To mitigate the increased cell susceptibility to SARS‐CoV‐2 infection caused by DNA damage, we treated cells with NMN and MDL‐800 to increase the DNA repair capacity. NMN supplementation can reinstate NAD^+^ levels which gradually decline in tissue and cell with age (Canto et al., 2012). NAD^+^ is an important coenzyme for redox reactions in energy metabolism and an essential cofactor for non‐redox NAD^+^‐dependent enzymes, including sirtuins and poly (ADP‐ribose) polymerases (PARPs), which function in metabolic pathways, DNA repair, chromatin remodeling, cellular senescence, and immune cell function (Covarrubias et al., 2021; Li et al., 2017). MDL‐800 is a SIRT6 activator and improves genomic stability by activating two DNA repair pathways—nonhomologous end joining (NHEJ) and base excision repair (BER; Chen, Chen, et al., 2020). We first detected DNA damage levels by comet assay after NMN and MDL‐800 treatment. Both agents' treatment result in noticeable decrease of DNA damage levels after 10 Gy IR treatment (Figure S7a). Homologous recombination (HR) and NHEJ are two major DNA repair pathways of DSB, which is one of the most toxic DNA lesions (Her & Bunting, 2018). We used the I‐SceI‐induced DSB in U2OS cells with NHEJ and DR‐GFP HR reporter systems to examine NHEJ and HR repair efficiency. As shown in Figure S7b, both NMN and MDL‐800 significantly increased NHEJ efficiency, and MDL‐800 have a slight effect on HR efficiency. Moreover, NMN and MDL‐800 treatment promoted γH2AX focal loss in Calu‐3 cells after IR, supporting their positive roles in DNA repair (Figure S7c). Next, we detected the function of NMN and MDL‐800 in cell susceptibility to SARS‐CoV‐2 infection. Both agents inhibited cell susceptibility to SARS‐CoV‐2 pseudovirus, as well as the expression of ACE2 and c‐Jun (Figure 6a). Furthermore, organoid (line 46) which isolated from a 55‐year‐old woman were used to examine the function of MDL‐800. NMN was not included in the experiment because it inhibited organoid differentiation. As expected, MDL‐800 reduced SARS‐CoV‐2 pseudovirus infection, as well as ACE2, γH2AX, and c‐Jun expression (Figure S8).

NMN and MDL‐800 regulate multiple cellular processes, including DNA repair, mitochondria quality, and chromatin structure (Chen, Chen, et al., 2020; Hong et al., 2020). To test whether NMN and MDL‐800 regulate viral entry through improving DNA repair capacity, we interrupted HR and NHEJ pathways through knockdown of RAD51 and 53BP1, which are indispensable for DNA repair after DOX‐induced DSB in Caco‐2‐Rluc cells expressing SacI (Figure S7b). Notably, inhibition of RAD51 and 53BP1 dramatically restricted the function of NMN and MDL‐800 in viral entry and ACE2 expression (Figure 6b), supporting the role of DNA repair in the function of these agents.

To examine the function of enhancing DNA repair capacity in SARS‐CoV‐2 infection in vivo, we fed older mice (12‐month‐old) with NMN by oral administration for 7 days and then infected the mice with SARS‐CoV‐2 mouse‐adapted virus. NMN treatment decreased the expression of γH2AX, which is consisted with previously reported (Li et al., 2017). Importantly, expression of ACE2 and p‐c‐Jun was also decreased in lung tissues of mice treated with NMN (Figure 6c). Moreover, NMN treatment decreased viral loads and tissue damage in mouse lung (Figure 6d–f). These results suggest that NMN treatment could alleviate SARS‐CoV‐2 infection and lung damage in vivo.

## 4 DISCUSSION

In the present study, we aimed to discover molecular mechanisms contribute to age‐associated differences in SARS‐CoV‐2 infection and explore new therapeutic targets. We found that DNA damage accumulated during aging increased the expression of ACE2 and SARS‐CoV‐2 infection through upregulation of the transcriptional factor c‐Jun. Inhibition of DDR pathways blocked the effect of DNA damage on both ACE2 expression and viral entry in vitro and in vivo. Moreover, enhancing DNA repair capacity through treatment of NMN or MDL‐800 alleviated SARS‐CoV‐2 infection and lung damage.

Although other receptors have been identified (Wang et al., 2021; Wang, Chen, et al., 2020), ACE2 is the major receptor for SARS‐CoV‐2 infection through its interaction with S protein of SARS‐CoV‐2 and thus is the crucial determinant for cross‐species transmission of the virus (Brown et al., 2021; Hoffmann et al., 2020). Structural and biochemical analyses identified a 211 amino acid region (amino acids 319–529) as the receptor binding domain (RBD) in C‐terminal domain of S protein (Shang, Ye, et al., 2020; Walls et al., 2020). After receptor engagement, specific proteases, including transmembrane protease serine protease 2 (TMPRSS2), cathepsin L, and furin, are required for the cleavage of S protein and trigger its fusogenic activity (Shang, Wan, et al., 2020). Single‐cell RNA sequencing data indicate that TMPRSS2 is co‐expressed with ACE2 in nasal epithelial cells, lungs, and bronchia, which explains some of the tissue tropism of SARS‐CoV‐2 (Lukassen et al., 2020; Sungnak et al., 2020). In the current study, we identified that DNA damage increased the expression of ACE2, but not that of TMPRSS2. Both DNA double‐strand break (DSB, induced by IR or etoposide treatment) and single‐strand break (SSB, induced by UV treatment) were tested in the current manuscript. A clean DSB was also generated through a doxycycline‐induced restriction endonuclease SacI system. Moreover, telomere damage induced by telomere attrition or uncapping also increased the expression of ACE2, which is consistent with a recent report (Sepe et al., 2021). These findings validate the regulation of DNA damage on ACE2 expression. Except for DNA damage, sex, genetic variants in the genes of the RAS system and other factors like the use of some anti‐hypertensive drugs, can affect the expression of ACE2. In particular, all these factors can affect the risk of infection of SARS‐CoV‐2 and determine the severity of the symptoms, which indicate the important roles of ACE2 levels in SARS‐COV‐2 infection and progression (Medina‐Enriquez et al., 2020). Although the exact mechanism remains unclear, we found that c‐Jun is required for DNA damage‐induced ACE2 expression and viral entry. c‐jun binds to ACE2 promoter and knockdown of c‐Jun inhibit DNA damage‐induced ACE2 expression and viral entry. c‐Jun has been reported to be activated by DNA damage and upregulated in older human and mouse tissues (Biteau et al., 2008; Suh, 2001), which is consistent with our findings. In addition, a positive correlation between DNA damage and the expression levels of p‐c‐Jun and ACE2 in human tissues was identified in this study. Therefore, we propose a DDR/c‐Jun/ACE2 signaling circuitry which may function in the age‐associated differences of SARS‐CoV‐2 infection.

Targeting viral entry is a promising antiviral strategy for virus. Take HIV for example, three FDA‐approved drugs against HIV, Maraviroc, Enfuvirtide, and Ibalizumab, are designed to target receptor or co‐receptor of HIV. These drugs can efficiently inhibit viral entry and improve clinical outcomes (Kitchen et al., 2008; Millham et al., 2020; Reuter et al., 2010). Thus, ACE2 and viral entry are ideal therapeutic target for COVID‐19. Some therapy approaches, like soluble ACE2 protein, Camostat, and EK1, improved symptoms and survival rate of mice infected with SARS‐CoV or SARS‐CoV‐2 (Hassler et al., 2021; Hoffmann et al., 2020; Xia et al., 2020; Zhou et al., 2015). Here, we identified that targeting DNA damage in vivo, either by using VE‐822 to inhibit DDR or by increasing the DNA repair capacity through treatment with NMN, reduced the expression of ACE2. More importantly, VE‐822 and NMN reduced viral loads and alleviated lung damage in older mice. These results indicate DNA damage is a potential target for COVID‐19.

DNA repair capacity decreases with age and contribute to the age‐associated accumulation of DNA damage (Chen, Geng, et al., 2020; Gorbunova et al., 2007; Li & Vijg, 2012). NMN is the precursor of NAD+, which is synthetized to replenish the consumption by NADase participating in physiologic processes including DNA repair, metabolism, and cell death (Hong et al., 2020). PARPs and sirtuins are two classes of NADase and play key roles in DNA repair (Fang et al., 2016; Kciuk et al., 2020; Ray Chaudhuri & Nussenzweig, 2017). In this manuscript, NMN treatment significantly increased NHEJ efficiency in vitro and dramatically inhibited DNA damage in vitro and in vivo (Figure S5a–c). Moreover, NMN treatment alleviated SARS‐CoV‐2 infection and lung damage in older mice (Figure 6d,e). The decline of NAD+ levels in the course of aging open the avenue for NAD+ supplement in anti‐aging therapy (Fang et al., 2016; Li et al., 2017). Interestingly, an NMN cocktail has been applied to treat older people with complicated SARS‐CoV‐2 infections and has resulted in rapid and dramatic clinical and laboratory improvement in a preliminary study (“Huizenga, Robert, Dramatic Clinical Improvement in Nine Consecutive Acutely Ill Elderly COVID‐19 Patients Treated with a Nicotinamide Mononucleotide Cocktail: A Case Series [August 17, 2020]. Available at SSRN: https://ssrn.com/abstract=3677428”). Due to the safety of NMN has been tested (Turner et al., 2021), NMN treatment may constitute a new approach for COVID‐19 therapies, especially in older adults. However, the limitation is that NMN likely does not repair DNA damage in senescent cells, such as the damage from dysfunctional telomeres or other persistent DNA damage, which protect from malignant transformation. Thus, NMN should be used in combination with senolytic drugs which clear senescent cells in our body to improve anti‐SARS‐CoV‐2 efficiency.

## AUTHOR CONTRIBUTIONS

L.C., C.C., Q.Y., and W.C. conceived the study, designed the experiments, and analyzed the data. R.J. designed and performed the experiments aided by X.Z. and S.X. C.N. and F.W. isolated organoid and performed related experiments. S.Z. and T.H. provided the clinical samples and performed related analysis. Z.Z. performed experiment with authentic virus. E.L. performed mice infection experiments aided by R.J. and F.W.J.W. provided help on comet assay and DNA repair reporter assay. L.C. drafted the manuscript and that was edited by S.T.

## FUNDING INFORMATION

L.C. was supported by the National Key Research and Development Program (2022YFC3600100), National Natural Science Foundation (82072717) and Beijing Nova Program (Z191100001119020). Q.Y. was supported by National Natural Science Foundation (81630067 and 81930078). Z.Z. was supported by National Natural Science Foundation (31900671).

## CONFLICT OF INTEREST

All authors declare that they have no competing interests.

## Supporting information


Figures S1–S8.

Table S1.

Table S2.

Table S3.

Table S4.

Table S5.

Table S6.

Appendix S1.

## Data Availability

All data needed to evaluate the conclusions in the paper are present in the paper and/or the Supporting Information. Additional data related to this paper may be requested from the authors.
