# Supplementary material for: DNA damage contributes to age‐associated differences in SARS‐CoV‐2 infection
Source: Aging Cell. 2022 Oct 18;21(12):e13729. doi: 10.1111/acel.13729 (PMC9741512; doi:10.1111/acel.13729)
Supplement: Supplementary file 1 — Figures S1–S8. [file ACEL-21-0-s006.pdf]

Fig. S1

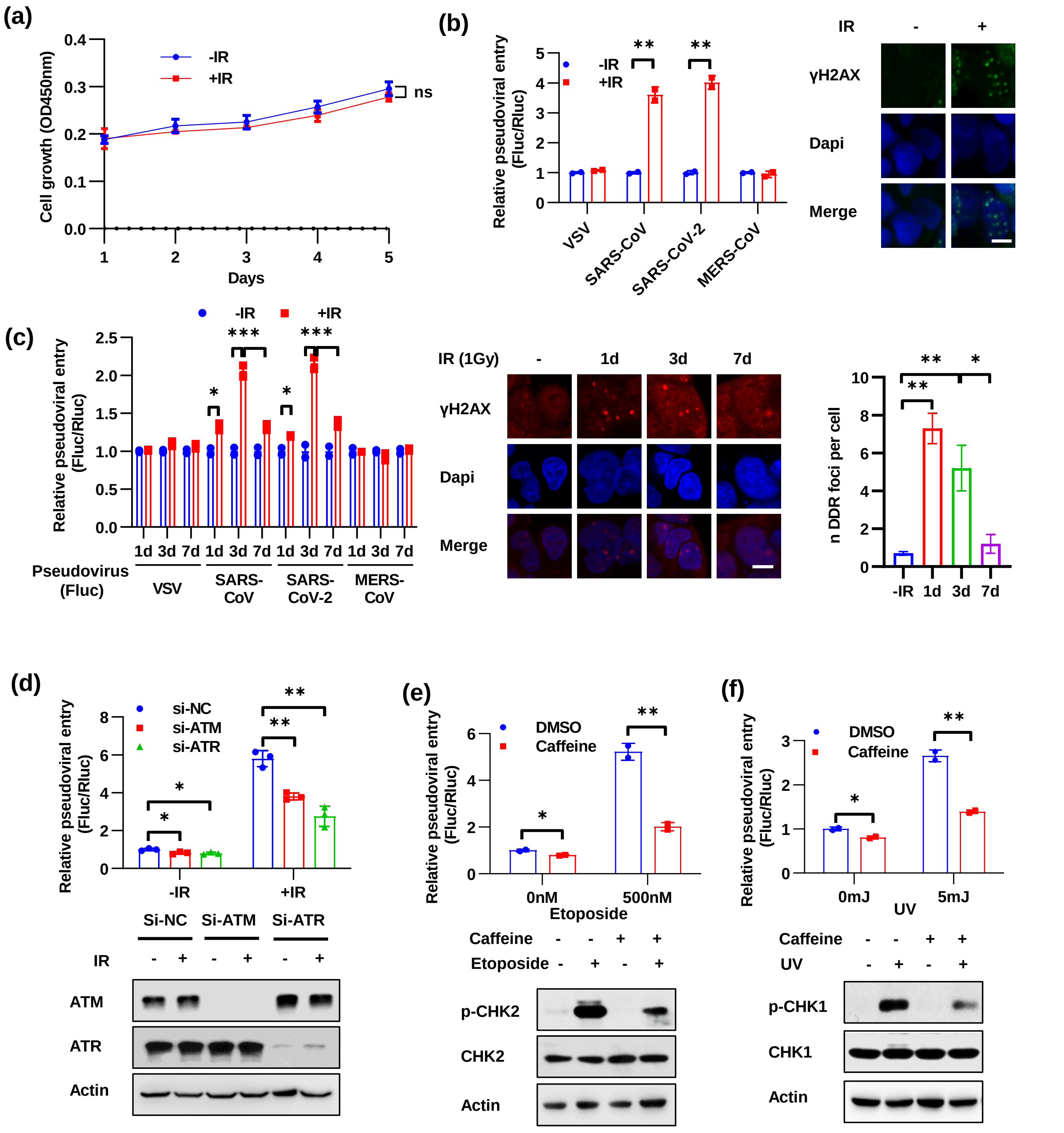
(a)
Cell growth (OD450nm)
-IR
+IR
ns
Days
(b)
Relative pseudoviral entry (Fluc/Rluc)
VSV
SARS-CoV
SARS-CoV-2
MERS-CoV
IR
γH2AX
Dapi
Merge
(c)
Relative pseudoviral entry (Fluc/Rluc)
1d 3d 7d 1d 3d 7d 1d 3d 7d 1d 3d 7d
Pseudovirus (Fluc)
VSV
SARS-CoV
SARS-CoV-2
MERS-CoV
IR (1Gy)
γH2AX
Dapi
Merge
n DDR foci per cell
-IR 1d 3d 7d
(d)
Relative pseudoviral entry (Fluc/Rluc)
si-NC
si-ATM
si-ATR
-IR
+IR
Si-NC
Si-ATM
Si-ATR
IR
ATM
ATR
Actin
(e)
Relative pseudoviral entry (Fluc/Rluc)
DMSO
Caffeine
0nM
500nM
Etoposide
Caffeine
Etoposide
p-CHK2
CHK2
Actin
(f)
Relative pseudoviral entry (Fluc/Rluc)
DMSO
Caffeine
0mJ
5mJ
UV
Caffeine
UV
p-CHK1
CHK1
Actin

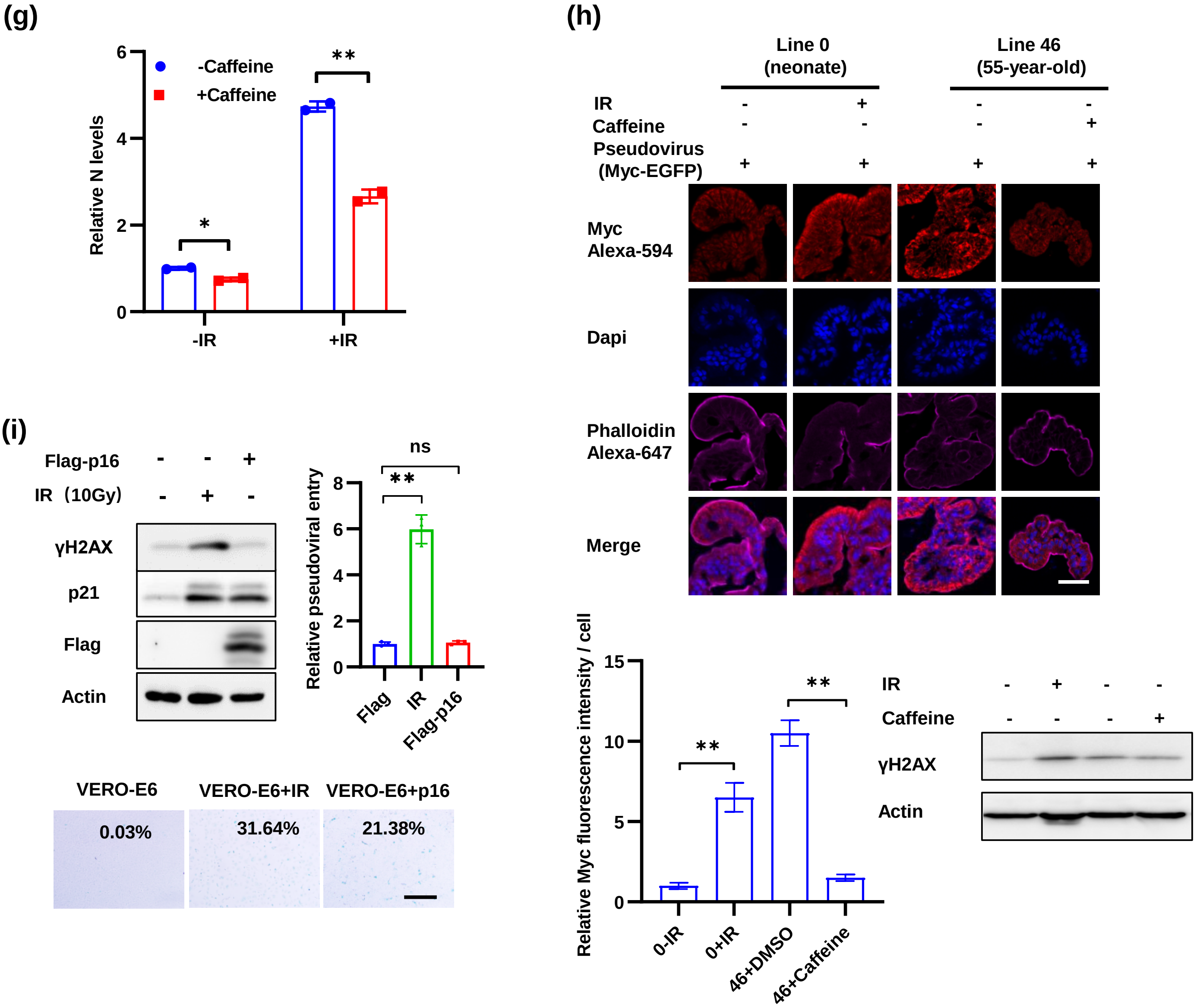
(g)
Relative N levels
-Caffeine
+Caffeine
*
**
-IR
+IR
(h)
Line 0
(neonate)
Line 46
(55-year-old)
IR
Caffeine
Pseudovirus
(Myc-EGFP)
Myc
Alexa-594
Dapi
Phalloidin
Alexa-647
Merge
Relative Myc fluorescence intensity / cell
0-IR
0+IR
46+DMSO
46+Caffeine
γH2AX
Actin
(i)
Flag-p16
IR（10Gy）
γH2AX
p21
Flag
Actin
Relative pseudoviral entry
ns
Flag
IR
Flag-p16
VERO-E6
VERO-E6+IR
VERO-E6+p16
0.03%
31.64%
21.38%

Fig. S1. DNA damage promotes SARS-CoV-2 entry through DDR. (**a**) Calu-3-Rluc cells treated with or without 10Gy IR were cultures in medium supplemented with 1% FBS and cell proliferation was monitored using CCK8 kit for 5 days. (**b**) Caco-2-Rluc cells on 3 day after 10Gy IR treatment were subjected to pseudotyped virus infection. Viral entry was analyzed by luciferase activity at 3 days after infection. DNA damage was detected by immunostaining with anti-γH2AX antibody. Scale bars, 10 μm. (**c**) Calu-3-Rluc cells on different days after 1Gy IR treatment were subjected to pseudotyped viral infection. Relative viral entry was analyzed by luciferase activity at 3 days after infection. DNA damage was immunostained with anti-γH2AX at 1, 3, 7 days after IR. Scale bars, 10 μm. For the quantification shown, γH2AX foci in around 100 cells per time were analyzed as mean ± SD. (**d**) Caco-2-Rluc cells transfected with ATM siRNA or ATR siRNA were incubated with SARS-CoV-2 pseudovirus and relative viral entry was analyzed by luciferase activity at 3 days after infection. Expression of ATM and ATR were detected by western blot with indicated antibodies. (**e,f**) Caco-2-Rluc cells were pre-treated with Caffeine (1 mM) for 12h and then subjected to etoposide **(e)** or UV **(f)** treatment, followed by incubation with SARS-CoV-2 pseudoviruses at 3 days after treatment. Viral entry was analyzed by luciferase activity at 3 days after infection. Expression of phosphorylated and total CHK1 (**f**) or CHK2 (**e**) were detected at 2h after etoposide or UV treatment by western blot. (**g**) Caco-2 cells pre-treated with Caffeine (1mM) and IR (10Gy) were infected with authentic SARS-CoV-2 virus for 6h. The relative virus levels were detected by qRT-PCR analysis of SARS-CoV-2 N expression. (**h**) Differentiated organoids isolated from a neonate (Line 0) and a 55-year-old woman (Line 46) were treated with Caffeine (1mM) for 12h or IR (10Gy) and then infected with SARS-CoV-2 pseudovirus containing Myc-EGFP. Organoids were collected at 3 days after infection and immunostained with indicated antibodies. Relative myc fluorescence intensity per cell were quantified by dividing fluorescence intensity by cell number from > 5 representative images and are presented as the mean ± SD. DNA damage was detected by western blot using anti-γH2AX antibody at 2 days after IR and Caffeine treatment. Scale bar, 50 μm. (I) VERO-E6 cells were treated with IR (10Gy) or transfected with Flag-p16 and then infected with SARS-CoV-2 pseudovirus 3 days later. Relative pseudoviral entry was detected by luciferase activity assay 3 days after infection. Protein expression and cell senescence were detected by western blot and SA-β-gal staining at 7 days after IR or p16 transfection. SA-β-gal data were quantified from > 5 independent cell counts up to a total of at least 200 cells and are presented as the mean percentage of positive cells. Scale bar, 100 μm. Data are representative of three independent experiments (d-f, h) or two independent experiments (a-c,g) (mean ± SD of three (d, i) or two (a-c, e) biological replicates. *$P < 0.05$, ** $P < 0.01$) .

Fig. S2

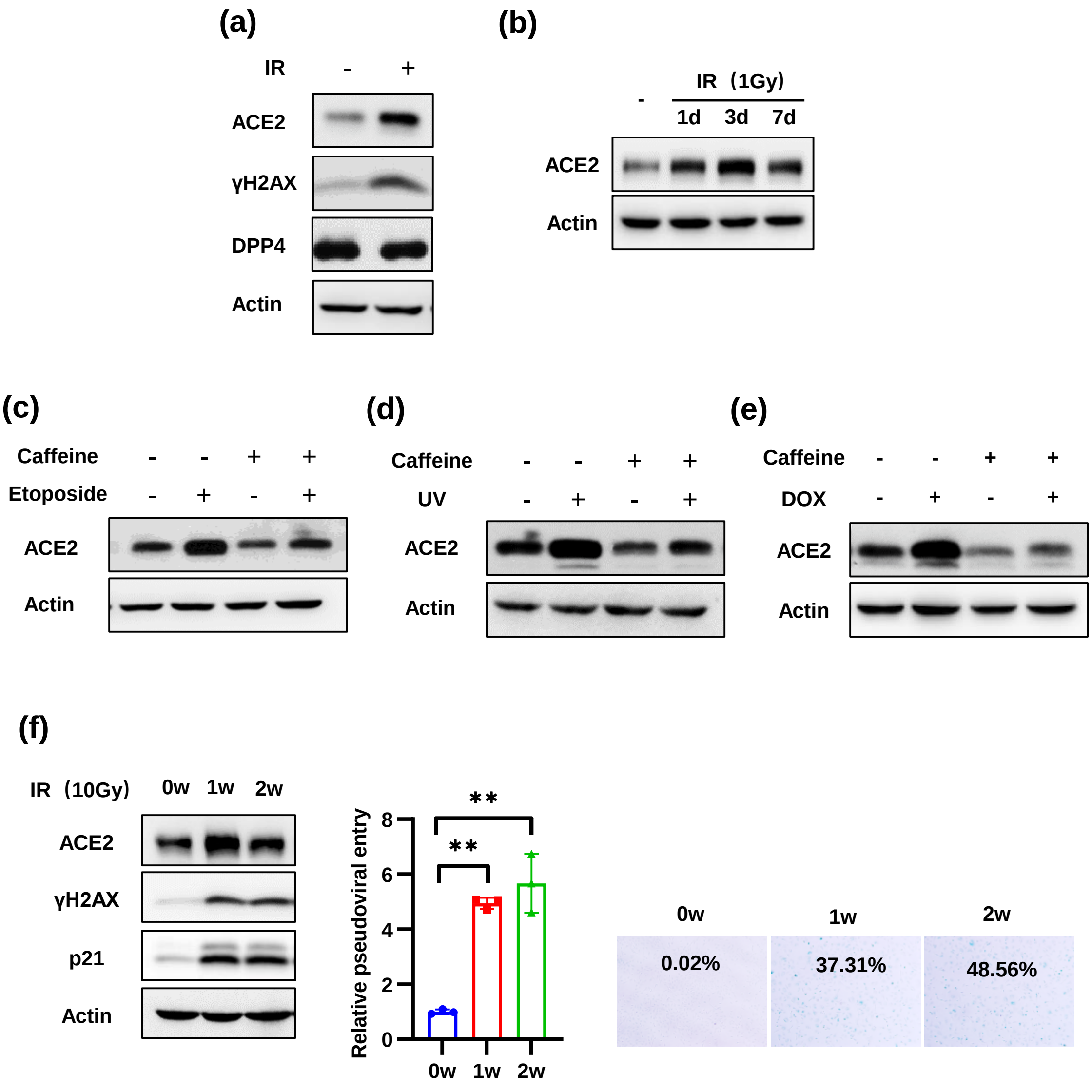

Fig. S2. DNA damage enhances ACE2 expression. (**a**) Calu-3 cells at 3 days after 10Gy IR treatment were subjected to western blot analysis using indicated antibodies. (**b**) Calu-3-Rluc cells at 1, 3, 7 days after 1Gy IR treatment were subjected to western blot analysis using the indicated antibodies. (**c,d**) Caco-2 cells were pre-treated with Caffeine (1 mM) for 12h and subjected to 500nM etoposide **(c)** or 5mJ UV **(d)** treatment, followed by western blot analysis using indicated antibodies at 3 days after treatment. (**e**) Caco-2 cells stably expressing dox-induced SacI were pre-treated with Caffeine (1mM) for 12h and subjected to DOX (2.5μM), followed by western blot analysis using indicated antibodies at 3 days after treatment. (F) VERO-E6 cells after 10Gy IR treatment were cultured for 1 week or 2 weeks and infected with SARS-CoV-2 pseudovirus. Protein expression, viral entry and cell senescence were detected. SA-β-gal data were quantified from > 5 independent cell counts up to a total of at least 200 cells and are presented as the mean percentage of positive cells. Scale bar, 100 μm. Data are representative of three independent experiments (a-f) (mean ± SD of three biological replicates (f)).

Fig. S3

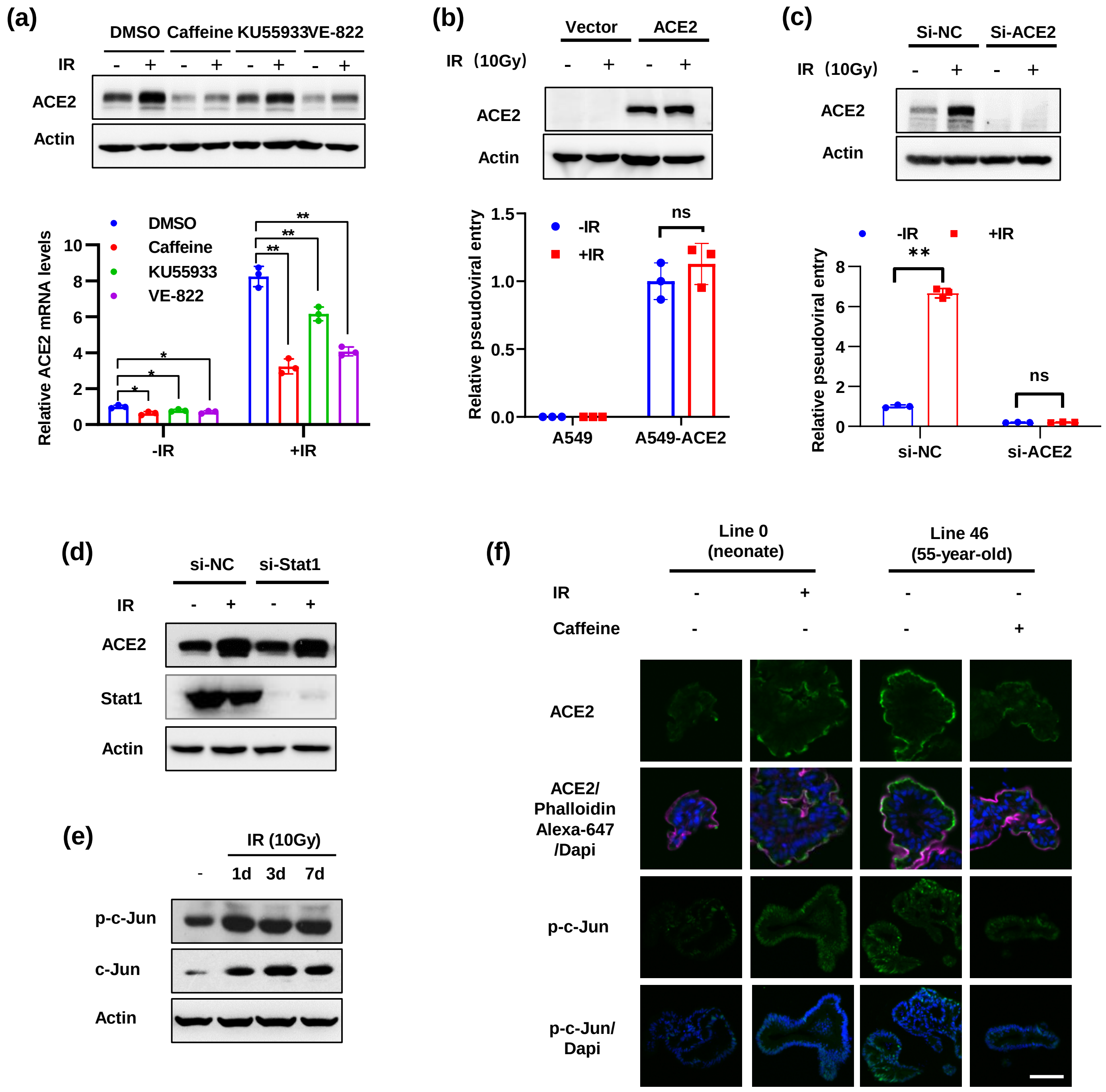

Fig. S3. DNA damage promotes the expression of ACE2 and c-Jun. (**a**) Calu-3-Rluc cells were pre-treated with Caffeine (1 mM), KU55933 (10 μM) or VE-822 (100 nM) for 12h and subjected to IR treatment (10Gy), followed by western blot analysis and qRT-PCR analysis of ACE2 at 3 days after IR treatment. (**b**) A549 and A549-ACE2 cells were treated with or without 10Gy of IR and infected with SARS-CoV-2 pseudovirus at 3 days after IR. ACE2 expression and pseudoviral entry were detected. **(c)** Caco-2 cells transfected with ACE2 siRNA were treated with 10Gy of IR, followed by infection with SARS-CoV-2 pseudovirus. Relative pseudoviral entry and ACE2 expression were detected. (**d**) Caco-2 cells transfected with Stat1 siRNA were treated with 10Gy of IR and then subjected to western blot analysis using the indicated antibodies at 3 days after IR treatment. (**e**) Calu-3-Rluc cells on different days after 10Gy IR treatment were subjected to western blot analysis using indicated antibodies. (**f**) Differentiated organoids treated with IR (10Gy) or Caffeine (1mM) were immunostained with the indicated antibodies. Scale bar, 50 μm. Data are representative of three independent experiments (a-e) or two independent experiments (f) (mean ± SD of three biological replicates (a-c)).

Fig. S4

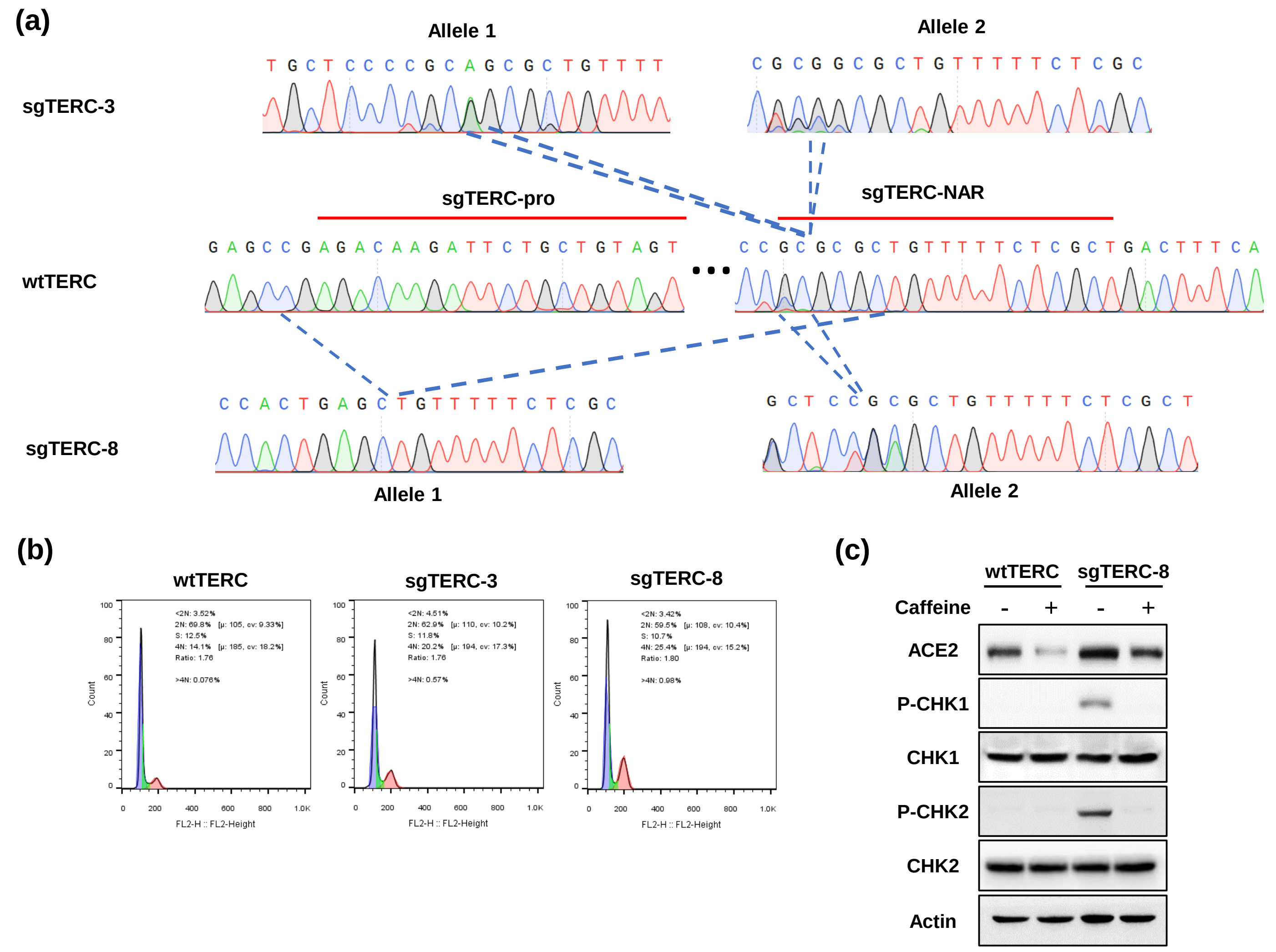

Fig. S4 Identification and character of sgTERC cells. (**a**) Sequencing of TERC knockout Calu-3 cells. Calu-3 cells stably expressing sgRNAs targeting TERC (sgTERC-pro and sgTERC-NAR) were constructed and dozens of single clones were selected and identified by DNA sequencing. Both alleles of two single clones (sgTERC-3 and sgTERC-8) were illustrated. Sequences of sgTERC-pro and sgTERC-NAR were marked in the sequences of wtTERC. (**b**) Cell cycle analysis of Calu-3 sgTERC single cells. (**c**) Cells were treated with Caffeine (1mM) and protein expression were examined using indicated antibodies.

Fig. S5

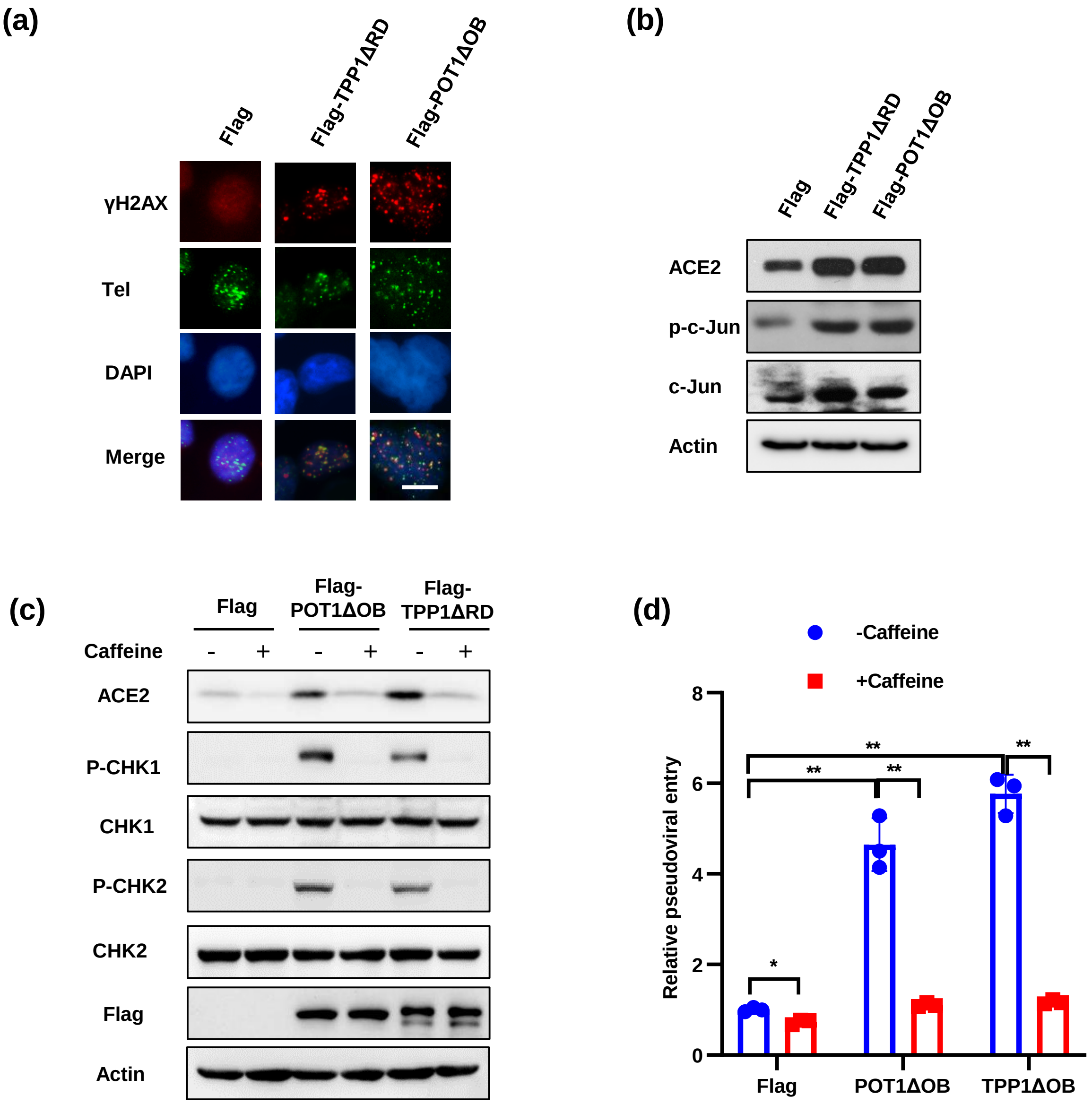

Fig. S5 Telomere uncapping promotes ACE2 expression and SARS-CoV-2 infection. (**a,b**) Caco-2 cells were transiently transfected with Flag-TPP1ΔRD or Flag-POT1ΔOB and telomere damage was detected by staining with anti-γH2AX antibody and Tel-Fish (**a**). Scale bar, 10 μm. Expression of ACE2, phosphorylated and total c-Jun levels were detected using indicated antibodies (**b**). (**c,d**) Caco-2 cells transfected with Flag-TPP1ΔRD or Flag-POT1ΔOB were treated with or without Caffeine (1mM) and followed by infection with SARS-CoV-2 pseudovirus containing luciferase. Protein expression (**c**) and viral entry (**d**) were analyzed. Data are representative of three independent experiments (a-d) (mean ± SD of three biological replicates (d)).

Fig. S6

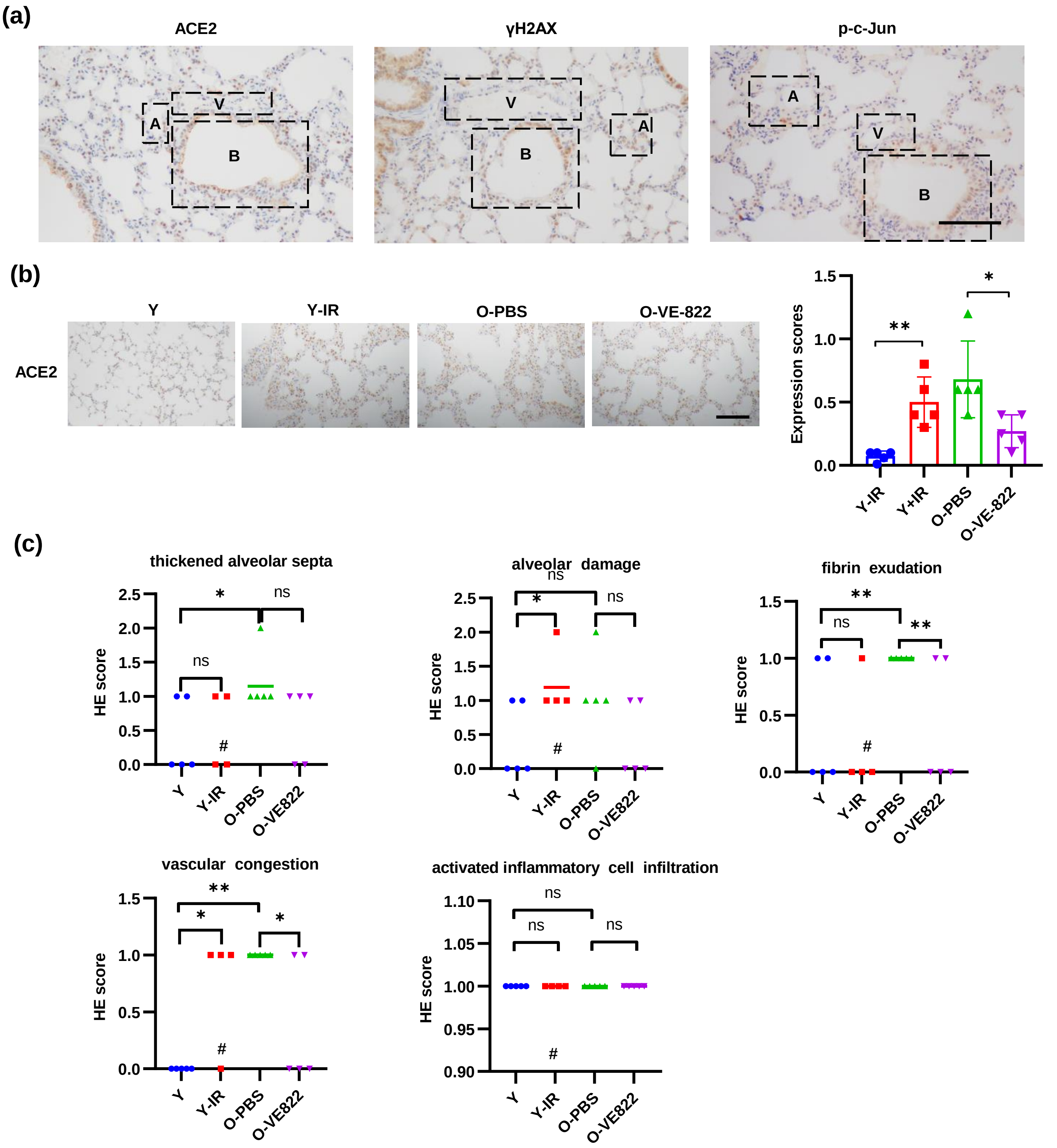

Fig. S6 DNA damage and DDR increase expression of ACE2 and lung damage in mice. (**a**) Immunostaining of ACE2, p-c-Jun and γH2AX in lung sections of mouse. B: bronchial airway, A: alveoli, V: blood vessel. Scale bar, 100 μm. (**b**) Expression of ACE2 was detected and analyzed in pulmonary alveoli region. (**c**) Data from semiquantitative analysis of histopathological changes of lung tissues after infection (HE scores) are presented as geometric means. #, one mouse died after anesthetization. $*P < 0.05$, $**P < 0.01$.

Fig. S7

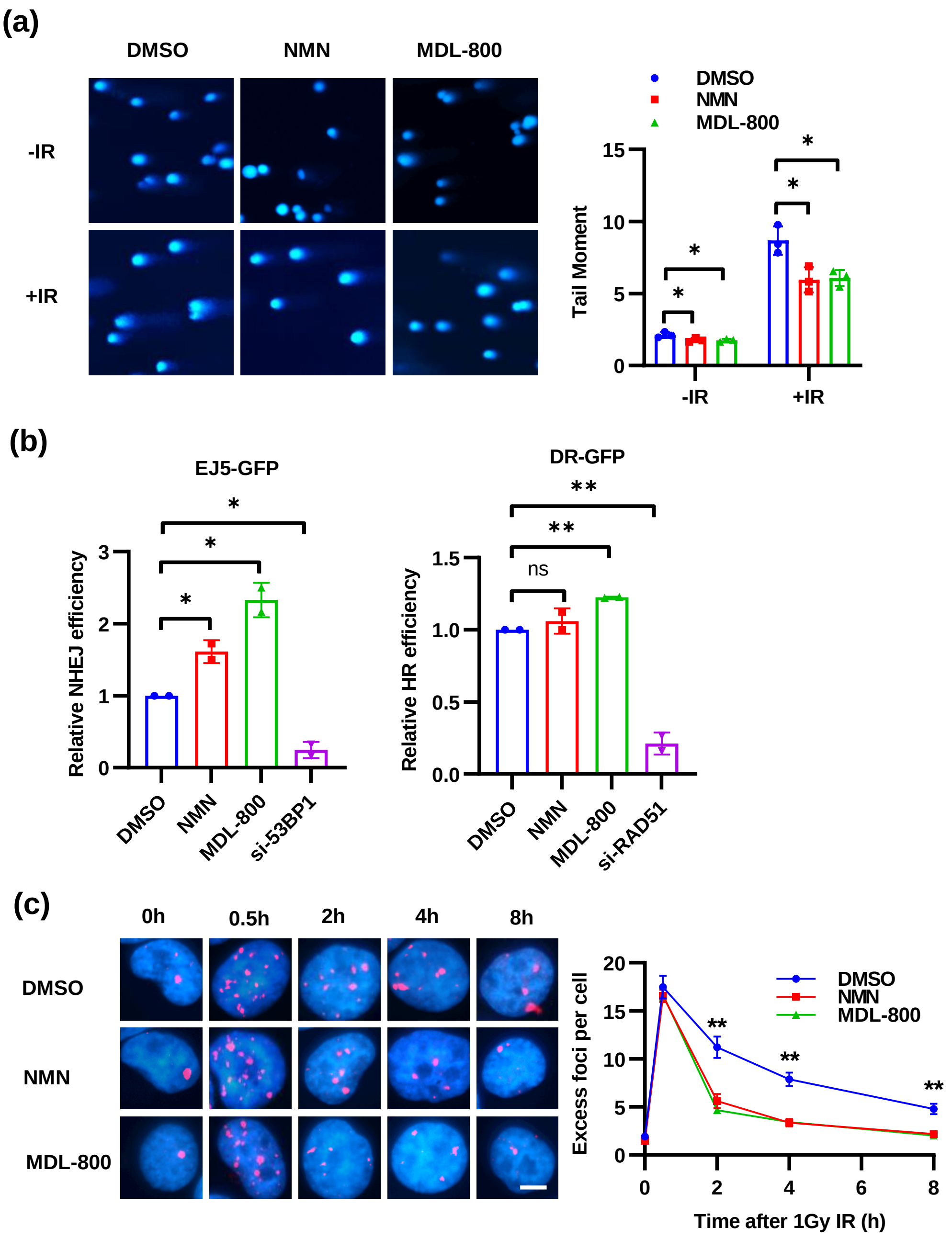

Fig. S7. NMN and MDL-800 inhibit DNA damage. (**a**) Alkaline comet assay was performed to assess DNA Damage. Caco-2 cells treated with DMSO, NMN (1 mM) or MDL-800 (20 μM) for 7 days and then subjected to 10Gy IR treatment and analyzed by alkaline comet assay at 4h after IR. Tail moments are quantified from at least 50 cells per group. Quantification data are represented as mean ± SD of three independent experiments. $*P < 0.05$. (**b**) U2OS NHEJ (EJ5-GFP) and HR (DR-GFP) reporter cell lines treated with NMN (1 mM) or MDL-800 (20 μM) for 7 days or transfected with indicated siRNA for 2 days were infected with lentivirus containing SceI. The cells were collected for flow cytometry analysis to determine NHEJ (top) and HR repair (bottom) efficiency. si53BP1 and siRAD51 were included as positive controls. Quantification data are represented as mean ± SD of two independent experiments. $*P < 0.05$, $**P < 0.01$. (**c**) Kinetics of γH2AX focal loss in Calu-3 cells after 1Gy IR. Calu-3 cells were treated with DMSO, NMN (1 mM) or MDL-800 (20 μM) for 7 days and then irradiated with 1Gy. The cells were isolated and stained for γH2AX antibody at indicated times. The average counts of foci are shown as mean ± SD of three independent fields with cell counts up to a total of at least 50 cells per group. $**P < 0.01$ versus corresponding DMSO treated cells.

Fig. S8

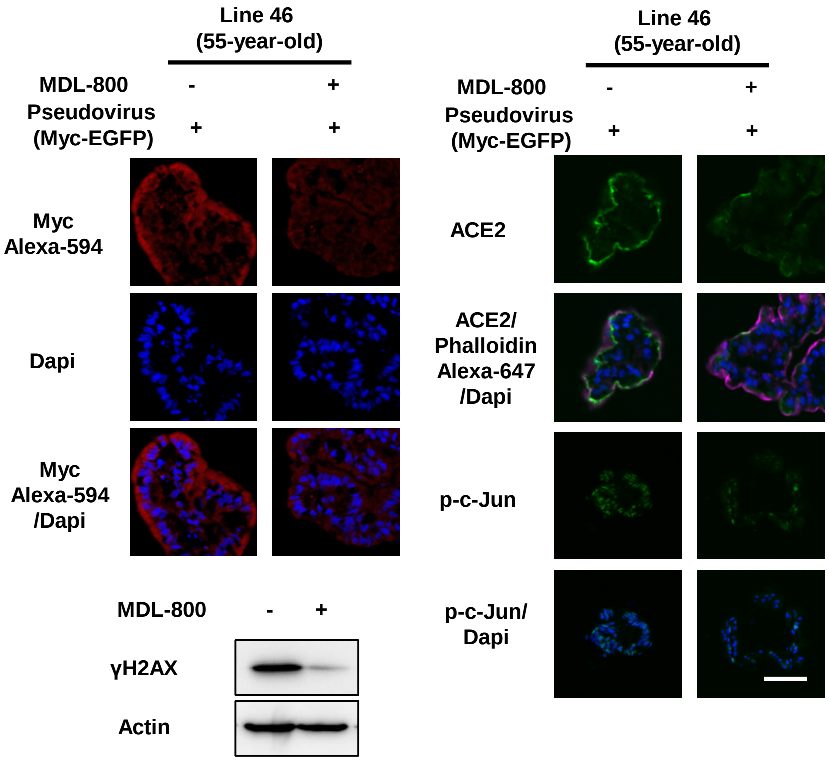

Fig. S8. MDL-800 inhibit SARS-CoV-2 pseudoviral entry in organoid. Differentiated organoids (Line 46) treated with MDL-800 (20 μM) for 3 days were infected with SARS-CoV-2 pseudovirus harboring Myc-EGFP. The organoids were harvested for immunostaining and western blotting with the indicated antibodies at 3 days post-infection. Scale bar, 50 μm.
