# Supplementary material for: DNA damage contributes to age‐associated differences in SARS‐CoV‐2 infection
Source: Aging Cell. 2022 Oct 18;21(12):e13729. doi: 10.1111/acel.13729 (PMC9741512; doi:10.1111/acel.13729)
Supplement: Supplementary file 8 — Appendix S1. [file ACEL-21-0-s004.docx]

**Supplementary Materials and Methods**

**Constructs and plasmids**

**Pseudotyped virus vectors**: Plasmids encoding SARS-CoV S glycoprotein (AAP13567.1), SARS-CoV-2 S glycoprotein (QHD43416.1) and MERS-CoV S glycoprotein (AFS88936.1) were constructed by inserting DNA fragments encoding codon-optimized SARS-CoV S protein lacking the last 19 amino acids (aa), encoding codon-optimized SARS-CoV-2 S protein lacking the last 19 amino acids (aa), MERS-CoV S protein lacking the last 16 aa into pCDNA3.0 between KpnI and EcoRI sites, respectively ([*1*](#_ENREF_1)). The VSV-G encoding plasmid and lentiviral packaging plasmids pLP1 and pLP2 were obtained from Addgene (Cambridge, MA). **Reporter vectors**: Renilla-luciferase (RLuc) lentivirus expression vector pHHLVX-EF1α-Rluc-puro was purchased from HedgehogBio Science and Technology Ltd. The lentiviral reporter plasmid that expresses Firefly luciferase (pCDH-Luc) was constructed by inserting DNA fragment encoding Firefly luciferase into pCDH-CMV-MCS-EF1-Puro (SBI, CD511B-1) between NheI and BamHI sites. The lentiviral plasmid expressing Myc-EGFP (pCDH-Myc-EGFP) was constructed by inserting DNA fragment encoding Myc-EGFP into pCDH-EF1-MCS-T2A-Puro (SBI, CD510A-1) between NheI and NotI sites. **pLVX-Tight-MNS-CMV-rtTA-T2A-puro**: The SacI coding sequence fused with nuclear localization sequence (NLS, PKKKRKV) was amplified from artificial synthesized DNA (synthesized by Biomed, Beijing, CHN) with Gflex polymerase (Takara) and cloned in frame with a Myc epitope into pCDH-EF1-MCS-T2A-Puro (SBI, CD510A-1) to get pCDH-EF1-Myc-NLS-SacI. CMV-rtTA and T2A-Puro-WPRE expression cassettes were amplified with Gflex polymerase from pLVX-tet on (Clontech) and pCDH-EF1-MCS-T2A-Puro (SBI, CD510A-1), respectively, and cloned into pLVX-Tight-Puro Vector (Clontech) between EcoRI and KpnI sites in head-to-tail orientation. Myc-NLS-SacI (MNS) fragment was PCR-amplified with Gflex polymerase and cloned into pLVX-Tight-CMV-rtTA-T2A-puro between EcoRI and NotI sites to get pLVX-Tight-MNS-CMV-rtTA-T2A-puro vector. **Other vectors**: The lentiviral plasmids Lenticas9-Blast (#52962) and Lentiguide-Puro (#52963) were obtained from Addgene and applied to knock-out of TERC. The lentiviral plasmids encoding deletion of the POT1-binding recruitment domain (244-337aa) of TPP1 (TPP1ΔRD) and lacking the first OB fold domain (1-126aa) of POT1 (POT1ΔOB) were constructed by inserting DNA fragments with a Flag tag at the N terminus into pCDH-EF1-MCS-T2A-Puro (SBI, CD510A-1) between NheI and NotI sites. All primers and codon-optimized gene sequences used in this study are listed in Supplementary Table 4.

**Senescence associated β-gal (SA-β-gal) staining**

The SA-β-gal staining was carried out according to the manufacturer’s instructions (Cell Signaling). Briefly, Cells were washed once with PBS, fixed with 0.5% glutaraldehyde in PBS for 15 min. After washed in PBS for 3 times, cells were stained in X-gal solution (100 mM sodium phosphate, 2 mM MgCl_2_, 150 mM NaCl, 0.01% sodium deoxycholate, 0.02% NP-40, 5 mM potassium ferricyanide, 5 mM potassium ferrocyanide, 1 mg/ml X-gal at pH 6.0) 6-24h at 37℃.

**Cell proliferation assay**

Calu-3-Rluc cells treated with or without IR were seeded onto 96-well plates (3×10^3^ cells/well), and cell proliferation was monitored using CCK8 according manufacturer’s instructions every 24 h for 5 days.

**RNA-seq**

RNA-seq assay was performed and analyzed by Wuhan Frasergen Bioinformatics Co. Ltd. Briefly, total RNA was extracted using the Trizol(Invitrogen, CA, USA), RNA purity and integrity was monitored by NanoDrop 2000 spectrophotometer (NanoDrop Technologies, Wilmington, DE, USA) and a Bioanalyzer 2100 system (Agilent Technologies, CA, USA). RNA contamination was assessed by 1.5% agarose gel. Oligo(dT)-attached magnetic beads were used to purify mRNA. Purified mRNA was fragmented into small pieces with fragment buffer at appropriate temperature. Then First-strand cDNA was generated using random hexamer-primed reverse transcription, followed by a second-strand cDNA synthesis and purified using AMPure XP Beads. Afterwards, A-Tailing Mix and RNA Index Adapters were added by incubating to end cDNA repair. The cDNA fragments obtained from previous steps were amplified by PCR, and products were purified by Ampure XP Bead, get the final library.

After the library was constructed, Qubit 2.0 was used for preliminary quantification, and the library was diluted to 1.5 ng/μL. Then Agilent 2100 BioAnalyzer was used to detect the insert size of the library. Qrt-pcr was used to accurately quantify the effective concentration of the library to ensure the quality of the library. Clean reads are obtained after filtering and compared to HISAT2 (V2.1.0) as reference genome. Using RSEM, the bowtie2 comparison results were used for statistics, and the number of Reads compared to each transcript of each sample was obtained, and FPKM (Fragments Per Kilobase Per Million bases) conversion was performed.

**Telomerase Repeat Amplification Protocol (TRAP) assay**

The Telomerase Repeat Amplification Protocol (TRAP) assay was performed to determine telomerase activity by using TRAPeze Telomerase Detection kit (Millipore) as previously described ([*2*](#_ENREF_2)). Briefly, cultured cell pellets were lysed with 1 × CHAPS lysis buffer for 30 min on ice. After centrifugation at 12000 rpm for 20 min, the concentration of supernatant proteins was determined by Bio-Rad Protein Assay Kit. Twenty nanograms of cultured cell lysates were used for TRAP assays. Chaps buffer was used as a negative control. The final reaction products were mixed with GelRed loading buffer (Generay Biotech) and run on a 10% polyacrylamide gel. The gel was exposed by Gel Image System (Tanon). Relative telomerase activity was calculated as the ratio of the intensity of the telomerase ladder over the intensity of the IC (internal control).

**Telomere length measurement**

The telomere length was measured according to the published method ([*3*](#_ENREF_3)). Genomic DNA was isolated from cells using KingFisher Flex DNA purification instrument (ThermoFisher) with MagMAX™ DNA Multi-Sample Ultra 2.0 Kit (ThermoFisher). After quantification, the genomic DNA was used for qPCR to detect the level of telomere (T) and single-copy gene 36B4 (S). The primers for telomere PCR were tel1b: 5-CGGTTT(GTTTGG)_5_GTT-3, used at a final concentration of 300nM, tel2b: 5-GGCTTG(CCTTAC)_5_CCT-3, used at a final concentration of 300nM. The primers for 36B4 PCR were 36B4u: 5-CAGCAAGTGGGAAGGTGTAATCC-3, used at a final concentration of 300nM, 36B4d: 5-CCCATTCTATCATCAACGGGTACAA-3, used at a final concentration of 500nM. 2×Mix (Qiagen) was used in qPCR reaction mixture with 9.2ng genomic DNA in each tube. qPCR was carried out on CFX-96 qPCR instrument (Bio-Rad). For telomere PCR, procedure includes 95℃ for 10min and 20 cycles of 95℃ for 15s, 56℃ for 1min. For 36B4 PCR, procedure includes 95℃ for 10min and 30 cycles of 95℃ for 15s, 60℃ for 1min. Relative T/S ratio of each sample was calculated and reflected relative telomere length. The T/S ratio for each sample was measured twice.

**Chromatin Immunoprecipitation (ChIP)**

ChIP assay was performed using EZ-Magna ChIP A/G Chromatin Immunoprecipitation Kit (Millipore) according to the manufacturer’s instructions. Briefly, cells treated with or without IR were cross-linked with 1% formaldehyde for 10 min, treated with glycine at a final concentration of 0.125 M for 5 min at room temperature, and lysed in lysis buffer for 15 min on ice. Nuclei were resuspended in nuclei lysis buffer, and the cross-linked DNA was sonicated for 10 min (with a 5 s on/off cycle) using a sonicator (SONICS). The supernatant was used for immunoprecipitation with anti-p-c-Jun or IgG and DNA purified from input or immunoprecipitants were subjected to qPCR. The following primers were used in qPCR reaction: c-Jun-pro-1: 5’- TGATCCTCTGTAGCCATGGGATCACA-3’, c-Jun-pro-2: 5’- TCCTCTCTTTGATCTGTGGCACTC-3’; c-Jun-dis-1: 5’- TACCATTTGACCCAGCAATCC-3’, c-Jun-dis-2: 5’- TGCTATTGTGAATAGTGCTGCA-3’; c-Fos-pro-1: 5’- GAGCAGTTCCCGTCAATCC-3’, c-Fos-pro-2: 5’- GTGAGCATTTCGCAGTTCCT-3’.

**Single-Cell Analysis**

Single cell data of nasal ciliated 2 cells ([*4*](#_ENREF_4)) were obtained from <https://www.covid19cellatlas.org/index.healthy.html>. Nasal data was extracted by using scanpy (v1.5.1) read_h5ad function and Seurat (v.3.1) was used for next step of analysis. Firstly, the data from different samples were integrated by IntegrateData function, and the cells were annotated according to the classification results in the previous article. Secondly, the cells were divided into two categories, ACE2+ group and ACE2- group, according to the presence or absence of ACE2 expression. Differential expression genes (logfc.threshold = 0.18) between ACE2+ cells versus ACE2- cells were performed enrichment analysis with Pathway Interaction Database (PID). The illustrations and statistical analysis were performed with R.

**HR-GFP and NHEJ-GFP reporter assays**

U2OS cells stably expressing HR reporter DR-GFP or NHEJ reporter (EJ5-GFP) were gifts from the Jiadong Wang laboratory at Peking University. For detecting NHEJ and HR repair efficiency in NMN and MDL-800 treated cells, cells were treated with NMN (1mM) or MDL-800 (20μM) for 7 days and then infected with lentivirus expressing I-SceI. Forty-eight hours later, cells were collected and subjected to flow cytometry analysis to determine percentages of GFP-positive cells.

**Single cell gel electrophoresis assay (alkaline comet assay)**

Caco-2 cells treated with NMN (1mM) or MDL-800 (20μM) for 7 days were irradiated with dose of 1 Gy and then further cultured for about 4h. The cells treated with or without IR were fixed in agarose on a slide and placed in a freshly configured cell lysate (2.5 mol/L NaCl, 100 mmol/L Na_2_-EDTA, 10 mmol/l Tris, 1% Triton X-100, 10% DMSO, pH 10) for 1h at 4℃ to dissolve the cell membrane. Then, the cells were placed in alkaline electrophoresis solution (0.3 mol/L NaOH, 1 mmol/L Na_2_-EDTA, pH 13) for 40 minutes at 4℃ and electrophoresis (25 V) for 30 minutes. Neutralizing solution (400mM Tris-Cl, ph7.5) was used to neutralize alkaline electrophoresis solution and the nuclei were counterstained with DAPI (1μg/ml) for 3 minutes. The images were captured by the fluorescence microscope (Olympus IX71) and analyzed by CaspLab version 1.2.3beta2. The results were expressed as tail moment. At least 50 cells were analyzed for each sample. Results were obtained from three independent experiments.

References

1. X. Ou, Y. Liu, X. Lei, P. Li, D. Mi, L. Ren, L. Guo, R. Guo, T. Chen, J. Hu, Z. Xiang, Z. Mu, X. Chen, J. Chen, K. Hu, Q. Jin, J. Wang, Z. Qian, Characterization of spike glycoprotein of SARS-CoV-2 on virus entry and its immune cross-reactivity with SARS-CoV. *Nature communications* **11**, 1620 (2020); published online EpubMar 27 (10.1038/s41467-020-15562-9).

2. L. Cheng, B. Yuan, S. Ying, C. Niu, H. Mai, X. Guan, X. Yang, Y. Teng, J. Lin, J. Huang, R. Jin, J. Wu, B. Liu, S. Chang, E. Wang, C. Zhang, N. Hou, X. Cheng, D. Xu, X. Yang, S. Gao, Q. Ye, PES1 is a critical component of telomerase assembly and regulates cellular senescence. *Sci Adv* **5**, eaav1090 (2019); published online EpubMay (10.1126/sciadv.aav1090).

3. R. M. Cawthon, Telomere measurement by quantitative PCR. *Nucleic Acids Res.* **30**, e47 (2002); published online EpubMay 15 (10.1093/nar/30.10.e47).

4. F. A. Vieira Braga, G. Kar, M. Berg, O. A. Carpaij, K. Polanski, L. M. Simon, S. Brouwer, T. Gomes, L. Hesse, J. Jiang, E. S. Fasouli, M. Efremova, R. Vento-Tormo, C. Talavera-Lopez, M. R. Jonker, K. Affleck, S. Palit, P. M. Strzelecka, H. V. Firth, K. T. Mahbubani, A. Cvejic, K. B. Meyer, K. Saeb-Parsy, M. Luinge, C. A. Brandsma, W. Timens, I. Angelidis, M. Strunz, G. H. Koppelman, A. J. van Oosterhout, H. B. Schiller, F. J. Theis, M. van den Berge, M. C. Nawijn, S. A. Teichmann, A cellular census of human lungs identifies novel cell states in health and in asthma. *Nat. Med.* **25**, 1153-1163 (2019); published online EpubJul (10.1038/s41591-019-0468-5).
